# Comparative proteomic analysis of human mesenchymal stromal cell behavior on calcium phosphate ceramics with different osteoinductive potential

**DOI:** 10.1016/j.mtbio.2020.100066

**Published:** 2020-06-24

**Authors:** Z. Othman, R.J.C. Mohren, B. Cillero-Pastor, Z. Shen, Y.S.N.W. Lacroix, A.P.M. Guttenplan, Z. Tahmasebi Birgani, L. Eijssen, T.M. Luider, S. van Rijt, P. Habibovic

**Affiliations:** a MERLN Institute for Technology-Inspired Regenerative Medicine, Department of Instructive Biomaterials Engineering, Maastricht University, Universiteitssingel 40, 6229 ER, Maastricht, the Netherlands; b The Maastricht Multimodal Molecular Imaging Institute (M4I), Division of Imaging Mass Spectrometry, Maastricht University, Universiteitssingel 50, 6229 ER, Maastricht, the Netherlands; c Department of Bioinformatics - BiGCaT, NUTRIM School of Nutrition and Translational Research in Metabolism Maastricht University, Universiteitssingel 50, 6229 ER, Maastricht, the Netherlands; d Laboratory of Neuro-Oncology and Clinical and Cancer Proteomics, Department of Neurology, Erasmus University Medical Center, Wytemaweg 80, P.O. Box 2040, 3000 CA, Rotterdam, the Netherlands; e Department of Psychiatry and Neuropsychology, MHeNs School for Mental Health and Neuroscience Maastricht University, Universiteitssingel 50, 6229 ER, Maastricht, the Netherlands

**Keywords:** Bone graft substitutes, Osteoinduction, Protein adsorption, Proteomics, Network analysis

## Abstract

In recent years, synthetic calcium phosphate (CaP) ceramics have emerged as an alternative to bone grafts in the treatment of large critical-sized bone defects. To successfully substitute for bone grafts, materials must be osteoinductive, that is, they must induce osteogenic differentiation and subsequent bone formation *in vivo*. Although a set of osteoinductive CaP ceramics has been developed, the precise biological mechanism by which a material directs cells toward osteogenesis and the role of individual chemical and physical properties in this mechanism remain incompletely understood. Here, we used proteomics to compare serum protein adsorption to two CaP ceramics with different osteoinductive potential, namely an osteoinductive β-tricalcium phosphate (TCP) and a non-osteoinductive hydroxyapatite (HA). Moreover, we analyzed the protein profiles of human mesenchymal stromal cells (hMSCs) cultured on these two ceramics. The serum protein adsorption experiments in the absence of cells highlighted the proteins that are highly abundant in the serum and/or have a high affinity to CaP. The extent of adsorption was suggested to be affected by the available surface area for binding and by the ion exchange dynamics on the surface. Several proteins were uniquely expressed by hMSCs on TCP and HA surfaces. Proteins identified as enriched on TCP were involved in processes related to wound healing, cell proliferation, and the production of extracellular matrix. On the other hand, proteins that were enriched on HA were involved in processes related to protein production, translation, localization, and secretion. In addition, we performed a separate proteomics analysis on TCP, HA, and two biphasic calcium phosphates with known osteoinductive potential and performed a clustering analysis on a combination of a set of proteins found to be enriched on osteoinductive materials with a set of proteins already known to be involved in osteogenesis. This yielded two protein networks potentially involved in the process of osteoinduction – one consisting of collagen fragments and collagen-related enzymes and a second consisting of endopeptidase inhibitors and regulatory proteins. The results of this study show that protein profiling can be a useful tool to help understand the effect of biomaterial properties on the interactions between a biomaterial and a biological system. Such understanding will contribute to the design and development of improved biomaterials for (bone) regenerative therapies.

## Graphical abstract

**Figure:**
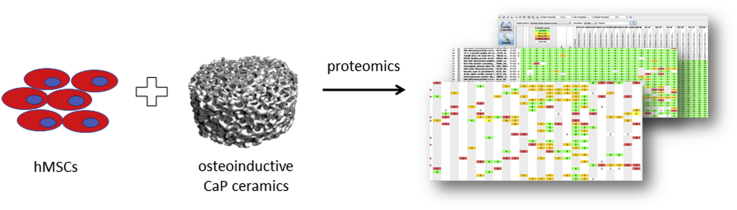

## 1 Introduction

Regenerative medicine comprises methods which harness and stimulate the body's own natural capacity to regenerate to restore damaged organs and tissues to normal function. As a result of an aging population and more active lifestyles, the demand for such treatments is increasing, as is the demand for the synthetic biomaterials which they often require to keep them affordable and widely accessible [1]. In particular, synthetic biomaterials have already shown some clinical success in the area of bone regeneration, where they substitute for either donor cadaveric bone or the patient's own grafted bone to allow the repair of large, critical-sized bone defects. Among the most important materials for this application are calcium phosphate (CaP) ceramics, which have a history of use in the treatment of craniomaxillofacial [2,3] and long bone defects [4] and for spinal fusion [5].

Initially, the chemical similarity between CaP ceramics and the mineral component of natural bone was exploited to produce biologically inspired biocompatible materials that were osteoconductive – able to serve as scaffolds for new growth of bone from a native bone surface [6]. In the past decades, CaP ceramics have been developed with the intrinsic ability to induce *de novo* bone formation *in vivo* even at ectopic implantation sites. This property, known as osteoinductivity, is considered imperative for the capacity of a biomaterial to regenerate large, clinically relevant bone defects [[7], [8], [9]]. So far, while not all CaP ceramics possess this ability, a range of CaP ceramics have been identified as osteoinductive in a variety of animal models [10]. Despite extensive research into defining the physical, chemical, and structural properties that determine whether a given CaP ceramic is osteoinductive, the exact mechanism is not yet completely understood. Chemical phase and surface chemistry, surface microstructural properties such as grain size, nanocrystal morphology, and microporosity, and the presence and architecture of ‘protected’ areas such as pores and channels in which local concentrations of calcium and inorganic phosphate ions can be modified have all been suggested as potentially playing a role in osteoinduction [[10], [11], [12], [13], [14], [15], [16]].

Several biological processes have been suggested to govern the process of osteoinduction by biomaterials. These include mechanobiological triggers, the role of endogenous osteoinductive growth factors such as bone morphogenetic proteins adsorbed or produced on the material surface, and the role of the inflammatory response to the implanted biomaterial [10,17,18]. A complicating factor in identifying the biological mechanisms responsible for CaP bioactivity is that ectopic bone formation is species dependent, where, in general, experiments in larger animals show a stronger osteoinductive potential [10]. However, experiments in these large animals provide limited possibilities to obtain mechanistic insights into the osteoinductive properties of ceramics. This is in part due to limitations in available assays, such as lack of species-specific antibodies, and because cost and ethical implications limit experiments in large animals to relatively few time points.

Recently, several *in vitro* studies have been performed on CaP ceramics with known osteoinductive potential, in an effort to unravel the biological mechanisms of osteoinduction. For instance, Groen et al [19] performed a transcriptomic analysis of MG63 human osteosarcoma cells cultured on 23 different biomaterials used in bone repair and regeneration, including several osteoinductive CaP ceramics. The resulting gene expression profiles were classified based on the structural and chemical properties of the biomaterials, and the authors were able to confirm the effect of osteoinductive CaP ceramics on bone morphogenetic protein 2 (BMP2) and transforming growth factor beta (TGF-β) signaling [19]. Similarly, Barradas et al. [20] performed DNA microarray and quantitative polymerase chain reaction (qPCR) analyses of gene expression of human mesenchymal stromal cells (hMSCs) cultured on a β-tricalcium phosphate (TCP) and a hydroxyapatite (HA) ceramic, similar to the ones used in this study. Markers related to osteogenic differentiation and bone extracellular matrix formation, including BMP2, osteopontin (OPN), bone sialoprotein, and osteocalcin (OC), were found to be upregulated in cells cultured on the osteoinductive TCP compared with those cultured on the non-osteoinductive HA [20]. In a series of studies on porous CaP ceramic scaffolds, Barba et al. [12,13] investigated the effect of different CaP ceramics on the expression of osteogenic markers by rat mesenchymal stromal cells (MSCs). They found that markers including BMP2, OC, and OPN were more upregulated on biomimetic calcium-deficient HA than on TCP or biphasic calcium phosphate (BCP) and suggest that this difference is due to the surface microstructure of the ceramics rather than their chemical properties [12,13].

Another study by Groen et al. [21] sought to link upregulation of genes to specific properties of materials. The study found an inverse relationship between the grain size of the material and the expression of four genes upregulated on osteoinductive materials – hyaluronan synthase 2 (HAS2); cell migration inducing protein, hyaluronan binding (CEMIP); activating transcription factor 3 (ATF3); and tenascin-C (TNC) – as well as a significant response by both CEMIP and TNC, as well as BMP2, to the levels of calcium and phosphate ions in the cell culture media. The upregulation of HAS2 and CEMIP suggests that hyaluronan synthesis may play an important role in osteoinduction [21]. A study by Tang et al [22] investigated the effect of CaP ceramics on gene expression *in vivo* in mice. The only ceramic which induced ectopic bone formation, a form of BCP, exhibited earlier and higher peaks in BMP2**,** osteoprotegerin, and bone morphogenetic protein receptor type IA expression. They also observed sequential activation of osterix (OSX) and type 1 collagen [22].

Finally, a range of studies by Davison et al [15,23] on CaP ceramics with different osteoinductive potential using osteoclasts explored the influence of material properties on the bioresorbability of the ceramics, another property which is important in the selection of materials for use in regenerative medicine. They found that the surface structure of ceramics was important for the differentiation and survival of osteoclasts [15,23].

Although these gene-level and functional studies provide important information, the studies investigating interaction between cells and CaPs on the protein level are still largely missing. Studies of adsorption of serum proteins on biomaterials and protein production by cells in contact with biomaterials can provide valuable information about the relationship between material properties and function [24], especially given that protein adsorption is the first interaction between a material and a biological system after implantation. Moreover, it is the production of proteins by adherent cells that ultimately determines cell fate and therefore the efficacy of regeneration [24,25].

In this study, we sought to further existing knowledge of cell behavior on osteoinductive biomaterials using two well-characterized CaP ceramics – an osteoinductive TCP and a non-osteoinductive HA [26]. Using label-free liquid chromatography-mass spectrometry (LC-MS/MS) analysis, we studied the adsorption of serum proteins and the protein profile of bone marrow–derived hMSCs cultured on TCP and HA. To further investigate the interactions between proteins involved in osteoinduction and their dependence on material properties, we performed a further series of cell culture experiments with hMSCs on a larger set of CaP ceramics, including two BCP ceramics, consisting of HA and TCP. Each BCP was sintered at a different temperature, and as a result, the two ceramics had identical bulk chemistry but different physical and possibly surface chemical properties and hence different osteoinductive potential [26,27]. LC-MS/MS analysis of hMSCs cultured on this larger set of materials was used to select proteins which were more abundant on osteoinductive than on non-osteoinductive CaPs. Computational methods were then used to generate a visualization of protein interaction networks involving both these proteins and a further set of proteins, derived from literature, which are known to be involved in cascades which play a role in osteogenic signaling.

## 2 Materials and methods

### 2.1 CaP ceramics

CaP ceramic particles with a diameter of 2–3 mm were produced as described previously [26,28]. Briefly, HA particles were produced from commercially available HA powder (Merck Eurolab BV, Amsterdam, the Netherlands) by dual-phase mixing as previously described [29] and sintered at 1250 °C for 8 h. TCP particles were prepared from TCP powder (Merck Eurolab) using the H_2_O_2_ method [30] and sintered at 1100 °C. BCPs were prepared from a combination of TCP powder (Plasma Biotal, Plasma Coating Ltd, Tideswell, UK) and calcium-deficient apatite powder (produced in-house) using the same H_2_O_2_ method and sintered at 1150 °C and 1300 °C to produce BCP1150 and BCP1300, respectively.

### 2.2 Cell culture

hMSCs were isolated from bone marrow aspirates of a single healthy donor who had given written informed consent. Cells were expanded on tissue culture polystyrene in proliferation medium consisting of α-MEM (minimal essential medium- alpha modification) (Gibco, Landsmeer, the Netherlands), 10% heat-inactivated fetal bovine serum (FBS) (Lonza, Basel, Switzerland), 0.2 mM ascorbic acid (Sigma, Zwijndrecht, the Netherlands), 2 mM l-glutamine (Gibco), 100 U/mL penicillin with 100 μg/mL streptomycin (Life Technologies, Bleiswijk, the Netherlands), and 1 ng/mL fibroblast growth factor (PhP105, Instruchemie, Delfzijl, the Netherlands) as described previously [31]. FBS was chosen instead of, for example, human serum, which may be clinically more relevant, as this is the standard protocol used in our laboratory and generally in the research field to culture and study hMSC behavior. Moreover, by using FBS, we could more easily distinguish between human proteins produced by hMSCs and bovine proteins adsorbed from serum in our proteomics analysis. Cells in culture were maintained at 37°C in a humidified atmosphere with 5% CO_2_. After reaching 80–90% confluence, cells at passage 2 were trypsinized, seeded on the ceramics at passage 3 at a density of 2 x 10^5^ per well containing 3 ceramic particles, and cultured for 8 h, 48 h, or 168 h in osteogenic medium (proliferation medium supplemented with 10 nM dexamethasone (Sigma)). The medium was refreshed every 3–4 days. While osteoinductive CaP ceramics have been shown to induce the osteogenic differentiation of hMSCs in basic and osteogenic cell culture medium [28], here we chose to culture in osteogenic medium to enable comparison with a previous study on hMSC behavior on these ceramics at the mRNA level [20], as well as to potentially increase the overall number of proteins produced by hMSCs when cultured on the ceramics.

### 2.3 LC-MS/MS analysis of protein adsorption on HA and TCP

Analysis of adsorbed serum proteins was performed as described previously [28,32] using a nano liquid chromatography system (Ultimate 3000, Dionex, Amsterdam, The Netherlands) coupled to a hybrid linear trap/orbitrap mass spectrometer (LTQ-Orbitrap-XL, Thermo Fischer Scientific, Bremen, Germany). Water, acetonitrile, and formic acid with ULC grade were obtained from Biosolve (Valkenswaard, the Netherlands). In brief, the HA and TCP ceramics (3 particles with a diameter of 2–3 mm) were immersed in 3 mL of osteogenic medium for 8 h, 48 h, or 168 h (n = 2 for each time point). The particles were washed twice with phosphate buffered saline, and the adsorbed proteins were lysed with Trizol (Invitrogen, Bleiswijk, the Netherlands). Proteins were precipitated using a methanol/chloroform mixture and resuspended in 20 μL of 0.1% (w/v) RapiGest (Waters Chromatography, Etten-Leur, the Netherlands) in 100 mM triethylammonium bicarbonate buffer at pH 7.5–8 to facilitate protein solubilization and cleavage. The samples were sonicated for 2 min using an ultrasonic cell disruptor at 70% power, heated to 95 °C for 5 min, then reduced with 1 μL of 100 mM dithiothreitol (DTT) solution for 30 min at 60 °C before cooling to room temperature, and alkylation with 1 μL of 300 mM iodoacetamide (IAA) solution in the dark for 30 min. The samples were incubated overnight with 2 μL of a 50 ng/mL trypsin solution at 37 C, before 0.5 μL 25% trifluoroacetic acid (TFA) was added to hydrolyze RapiGest and quench the reaction. After adjusting their pH to 2, the samples were centrifuged at 20,800×*g* for 20 min.

Samples were loaded onto a C18 trap column (PepMap C18, 300 μm ID x 5 mm length, 5 μm particle size, 100 Å pore size; Dionex) and desalted for 10 min with 0.1% TFA. The trap column was then switched online with an analytical column (PepMap C18, 75 μm ID x 150 mm length, 3 μm particle size, 100 Å pore size; Dionex) and peptides eluted using a binary gradient of 0–25% solvent B in solvent A over 120 min, followed by 25–50% solvent B over 60 min, where solvent A was 2% acetonitrile and 0.1% formic acid in water and solvent B was 80% acetonitrile and 0.08% formic acid in water. A data-dependant acquisition method was used, with an initial high-resolution survey scan from mass to charge ratio (*m/z) of* 400 to 1800 with the value of the automatic gain control (AGC) set to 106, resolution of 30,000 at m/z 400, and lock mass set to 445.120025 u (protonated (Si(CH_3_)_2_O)_6_). Based on this survey, the 5 most intense ions were consecutively isolated (AGC target set to −10^4^) and fragmented by collision-activated dissociation applying 35% normalized collision energy in the linear ion trap.

### 2.4 Initial LC-MS/MS analysis of proteins by hMSCs cultured on ceramics

hMSCs were cultured on HA and TCP particles as described previously. After 8 h, 48 h, or 168 h of culture (n = 4 for each time point), the constructs were placed in 5 M urea (GE Healthcare, Eindhoven, the Netherlands) with 50 mM ammonium bicarbonate (Sigma), and the cells were lysed using 3 freeze-thaw cycles (warm water and liquid nitrogen). The lysate was reduced with 20 mM DTT for 45 min and alkylated with 40 mM IAA for 45 min in the dark. To terminate the alkylation, 20 mM DTT was added to the lysate again. The lysate was digested with a mixture of LysC and trypsin (Mass spec grade; Promega, Leiden, the Netherlands) at a 1:25 enzyme to protein ratio. After 2 h of digestion at 37 °C, the lysate was diluted five-fold with 50 mM ammonium bicarbonate and further digested at 37 C overnight, before digestion was terminated with the addition of 1% formic acid.

LC-MS/MS was performed using an Ultimate 3000 Rapid Separation ultra high performance liquid chromatography (UHPLC) system (Dionex) coupled to a Q Exactive HF mass spectrometer (Thermo Scientific, Bremen, Germany). Peptide samples were desalted on an online C18 trapping column and then separated on an Acclaim PepMap C18 analytical column (75 μm ID x 150 mm length, 2 μm particle size, 100 Å pore size) using a 90-min linear gradient from 5% to 35% acetonitrile with 0.1% formic acid and a flow rate of 300 nL/min. Mass spectrometry was performed in data-dependent acquisition mode with a full MS scan from *m/z* 375 to 1500 at a resolution of 120,000, followed by MS/MS scans of the 15 most intense ions at a resolution of 30,000.

### 2.5 Protein identification and statistical analysis

Protein identifications from MS/MS results were validated using Scaffold (Proteome Software Inc., Portland, OR, USA) to verify peptide identifications using the X! Tandem database searching program [33,34]. Scaffold was then used to probabilistically validate peptide identification using PeptideProphet [35] and derive corresponding protein probabilities using ProteinProphet [36,37].

Serum protein adsorption on the two ceramics was analyzed using a Fischer exact test applied using Scaffold. Any non-bovine proteins identified were ignored, as the only source of proteins (other than contamination by e.g. human keratin) in these cell-free experiments was the FBS in the medium. Proteins were identified as being adsorbed on both ceramics if they were detected in both replicate samples for both ceramics and were identified as being adsorbed only on TCP if they were detected on both TCP replicates and on neither HA replicate.

For the initial analysis of proteins produced by cells cultured on HA and TCP, protein quantitation was performed in Scaffold using the default label-free quantitation in Proteome Discoverer, version 2.2, software (Thermo). Peptide precursor intensities, normalized using total peptide amount, were used to determine peptide abundances. Protein ratios were calculated based on pairwise peptide ratios, and background based analysis of variance (ANOVA) was used for hypothesis testing. Adjusted p-values were calculated using the Benjamini-Hochberg false discovery rate (FDR) correction for multiple tests. The data were processed, and principal component analysis (PCA) was performed using the MaxQuant [38] and Perseus [39] computational platforms.

For the analysis of proteins produced by cells cultured on the larger set of ceramics, raw data were preprocessed using Progenesis, version 4.0 (Nonlinear Dynamics, Newcastle upon Tyne, UK), and peptides were identified and assigned to proteins using Bioworks, version 3.2 (Thermo Fisher). The resulting file was submitted to Mascot v2 (Matrix Science, London, UK) for identification using the UniProt *Homo sapiens* database (release 2013_07, 20,265 sequences). Only proteins with at least two unique peptides (Mascot ions score >25, corresponding to a peptide probability cutoff of 0.01) were accepted as identifications. Mascot search results were imported back into Progenesis 4.0 to link identified peptides to their detected abundances, which were normalized to the total ion current.

### 2.6 Gene Ontology and pathway analysis

Gene Ontology (GO) terms and established biological pathways can be helpful in identifying which biological process sets of differentially expressed proteins could be involved in. Upregulated and downregulated proteins were selected from the data set collected in Section 2.4, proteins were considered to be upregulated or downregulated if at least a 1.3-fold change was detected with an FDR-corrected p-value less than 0.05. The Panther GO tool, version 14.1 [40,41], was used to perform enrichment analysis using the GO [42] and pathway analysis using the Reactome Pathway Database [43].

### 2.7 Network analysis

The data set whose analysis is described in this section was produced as part of our previously published experimental study [28]. Briefly, cells cultured on HA, TCP, BCP1150, and BCP1300 ceramics for 8 h, 48 h, and 168 h (n = 3 for each time point) were trypsinized according to a standard procedure. Samples (20 μL) of trypsinized cells were processed using the same method as in Section 2.3.

Network visualizations were generated using version 11.0 of the STRING protein query database [44] within Cytoscape, version 3.7.1 [45], to find interactions based on a range of evidence channels including text mining, databases of known associations such as KEGG [46], high-throughput experimental data from the ArrayProspector server [47], and cooccurrence based on similar patterns of mRNA expression. Two sets of proteins were analyzed in this way – the first was the set of the ‘top 50’ proteins found to be produced differentially on osteoinductive and non-osteoinductive ceramics by LC-MS/MS analysis [28]. The second set consisted of the initial 50 proteins plus a further 39 proteins known from literature to be involved in the bone morphogenetic protein (BMP) or Wnt signaling cascades, which have been shown to play a role in osteogenic signaling [48]. The proteins in each set are listed in Tables S19 and S20.

Protein interactions were filtered based on the combined interaction score from STRING, with a threshold value of 0.9 [[49], [50], [51]]. A clustering analysis was then performed using the ClusterOne application [52] with a minimum size of 3 and automated minimum density, and only clusters with p-value <0.05 were considered viable.

## 3 Results and discussion

### 3.1 Properties of ceramics

The CaP ceramics used in this study have previously been characterized both in terms of their physicochemical and structural properties and in terms of their osteoinductive potential [26,[53], [54], [55], [56]]. The HA and TCP ceramics comprised distinct CaP chemical phases, both ceramics were pure, with a maximum of 5 wt% phase impurity. The two BCP ceramics consisted of the same combination of phases – both were 20 wt% TCP and 80 wt% HA. It should be noted that the chemical composition of the four ceramics was assessed on the bulk material and that no specific analyses of the surface chemistry were performed. Previous studies have shown that upon sintering in air, phosphate evaporation occurs on the surface of CaP ceramics, leading to the formation of a calcium-rich phase (often CaO), which in turn can convert to Ca(OH)_2_ and eventually CaCO_3_ upon exposure to ambient air [[57], [58], [59], [60], [61], [62]] The extent of this process and the spatial organization of the formed layer are dependent on the bulk composition of the ceramic and the sintering conditions. While a combination of x-ray photoelectron spectroscopy and time-of-flight secondary ion mass spectrometry has been suggested before to be a useful tool in analyzing the composition of the surface [63], the extremely small thickness of the layer hampers the reliability of these techniques and presents the need for the more advanced techniques such as atomic probe tomography and magic angle spinning nuclear magnetic resonance. While we did not perform a detailed analysis of the surface chemistry of the ceramics, the possible effects of the differences in surface chemical composition on the protein adsorption and production by hMSCs cannot be fully excluded.

All ceramics were highly porous with comparable macroporosity, but their microporosity, defined as the volume percentage of pores with diameter less than 10 μm, varied significantly, with values more than 40% for the osteoinductive TCP and BCP1150 but less than 10% for the non-osteoinductive HA and BCP1300. In consequence, the specific surface area of the non-osteoinductive ceramics (0.1–0.2 m^2^/g) was about an order of magnitude lower than that of the osteoinductive ceramics (1–1.2 m^2^/g).

The osteoinductive potential of ceramics similar to that used here has been investigated *in vivo* using a range of animal models. All four ceramics have been implanted intramuscularly for 12 weeks in dogs [26] and goats [53], as well as subcutaneously in mice for 6–7 weeks [54], while subsets of them have been implanted intramuscularly for 8 weeks in mice [55] and subcutaneously for 12–24 weeks in dogs [56]. In addition, *in vitro* studies on the gene level have shown an increased expression of osteogenic markers such as OPN and BMP2 in hMSCs cultured on TCP compared with those cultured on HA [20,26].

### 3.2 Serum protein adsorption on HA and TCP

**Fig. 1:**
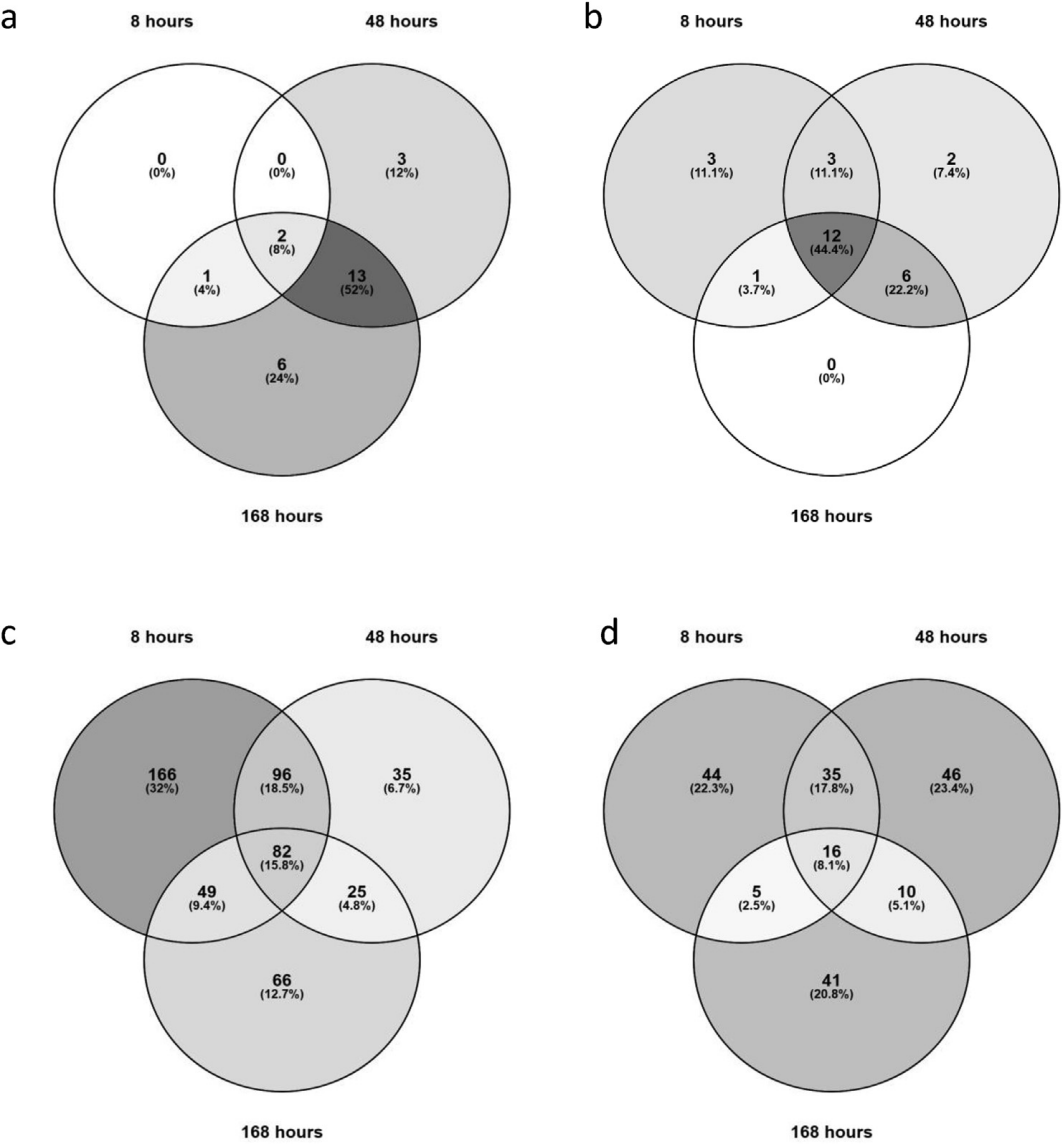
Venn diagrams showing proteins shared between different data sets. (a) Proteins exclusively adsorbed on TCP after 8, 48, and 168 h (Tables S1–3). (b) Proteins adsorbed on both HA and TCP after 8, 48, and 168 h (Tables S4–6). (c) Proteins more abundant in cells cultured on HA than in cells cultured on TCP after 8, 48, and 168 h (Tables S7–9). (d) Proteins more abundant in cells cultured on TCP than in cells cultured on HA (Tables S10–12). Diagrams produced using Venny: Oliveros, J.C. (2007–2015) Venny. An interactive tool for comparing lists with Venn's diagrams. https://bioinfogp.cnb.csic.es/tools/venny/index.html.TCP, β-tricalcium phosphate; HA, hydroxyapatite.

Adsorption of proteins from the biological environment on the surface of a biomaterial plays an important role in cell adhesion and in the instruction of other cellular processes such as proliferation and differentiation [24,25]. In the specific case of osteoinduction, it has been suggested, though not conclusively proven, that the entire process may be governed by initial protein adsorption [7,10,64]. Therefore, we first set out to understand binding of serum proteins to the surface of HA and TCP. To this end, particles of HA and TCP were immersed in osteogenic cell culture medium in the absence of cells for 8, 48, or 168 h. The profiles of bovine proteins adsorbed on the ceramic surfaces were analyzed by LC-MS/MS. Serum proteins were shown to adsorb to both CaP ceramics. In total, 28 proteins were found to be exclusively adsorbed on TCP – 3 after 8 h, 18 after 48 h, and 22 after 168 h, including 15 of the 18 found to be exclusively adsorbed after 48 h (Fig. 1a, Tables S1–3). No proteins were found to be exclusively adsorbed on HA.

The adsorption of proteins on a solid surface is controlled by a range of physical and chemical properties of the material [65], which has previously been shown for different CaP ceramics [66]. HA and TCP differ in a range of properties including chemical phase, microporosity and specific surface area, and consequently also in their degradation behavior [26]. All of these properties therefore need to be considered as possible factors in the observed differences in protein adsorption between the two materials. Importantly, it should be noted that there is a 12-fold difference in specific surface area between the materials and that the amount of ceramic used for the studies was not normalized for surface area. Therefore, the TCP particles presented a larger available surface for protein adsorption. We chose not to do the normalization, because also *in vivo*, when osteoinductive or bone regenerative potential are tested, the same amount of materials is implanted, not taking differences in specific surface area into account. It is suggested that this difference in the specific surface area is the main reason for a set of proteins to be solely observed on TCP, rather than the binding selectivity, considering that both ceramics have the same chemical nature. Among these proteins, worth mentioning are members of the complement system [67] (component C3, C7, C9), observed at the different time points, along with factor XIIa inhibitor, involved in regulation of complement activation and coagulation pathway [68], and vitamin D–binding protein, which has previously been shown to affect processes related to bone resorption by regulating vitamin D availability [69,70].

In addition to the proteins exclusively adsorbed on TCP, 28 bovine proteins were identified that were adsorbed on both ceramics (Fig. 1b, Tables S4–6), of which 4 showed significant difference in abundance between the two ceramics (Table 1, Table 2). As expected, the most abundant proteins adsorbed on both ceramics were albumin and alpha-2-HS-glycoprotein (fetuin A), both known to be present in relatively high amounts in (bovine) serum [71,72]. Although a higher abundance of bovine serum albumin was observed on TCP than on HA, the difference was not significant. In contrast, significantly more fetuin A was observed on TCP than on HA at both 48 h and 168 h (15- and 17-fold, respectively).

**Table 1:** Proteins significantly more abundantly adsorbed on TCP than on HA after 48 h.

Protein name	Gene name	Fisher exact test (*P*-value)	Fold change TCP/HA
Alpha-2-HS-glycoprotein (fetuin A)	AHSG	<0.00010	15
Matrix gla protein	MGP	0.00014	0.9
Antithrombin III	SERPINC1	0.044	2

TCP, β-tricalcium phosphate; HA, hydroxyapatite.

**Table 2:** Proteins significantly more abundantly adsorbed on TCP than on HA after 168 h.

Protein names	Gene name	Fisher exact test (*P*-value)	Fold change TCP/HA
Alpha-2-HS-glycoprotein (fetuin A)	AHSG	<0.00010	17
Matrix gla protein	MGP	<0.00010	0.7
Cytochrome C	CYC	0.00032	0.7

TCP, β-tricalcium phosphate; HA, hydroxyapatite.

As extensively reviewed, for example, in the study by Brylka et al [71], fetuin A has been described as a prototypic systemic inhibitor of mineralization. Fetuin A–deficient mice show impaired growth of their long bones and premature growth plate closure [73]. *In vitro*, fetuin A has been suggested to mediate proper collagen I mineralization [74]. In serum and bone, fetuin A has been shown to bind to TGF-β/BMP cytokines and to block the TGF-β signaling in osteoblastic cells [75]. An important property of fetuin A consistently been confirmed on the molecular level, both *in vitro* and *in vivo*, is its high affinity to bind CaP, making it one of the most abundant non-collagenous proteins in bone. Given that no cells were present in our protein adsorption experiment, the significantly higher adsorption of fetuin A to TCP than to HA can only be due to differences between the two ceramics. As mentioned before, TCP has a significantly higher specific surface area than HA, allowing for more fetuin A to adsorb. Moreover, an effect of this higher specific surface area, along with the difference in the chemical phase between TCP and HA, is a more pronounced depletion of calcium and inorganic phosphate ions from the cell culture medium by the TCP [28] which in turn may result in enhanced coprecipitation of fetuin A.

In contrast to fetuin A, matrix Gla protein (MGP) was shown to be slightly more abundant on HA than on TCP (up to 1.8- and 1.4-fold after 48 and 168 h, respectively). MGP, similar to fetuin A, has been previously described to be involved in regulation of (ectopic) bone formation. This low-molecular-weight protein is expressed mainly in chondrocytes and vascular smooth muscle cells and has been shown to act as a physiological inhibitor of ectopic tissue calcification [76,77]. MGP is a member of mineral-binding Gla protein family [78], which explains its binding to the two CaP ceramics here. Although without further research it remains speculative why a slightly higher abundance was observed on HA than on TCP, an earlier study has shown that the binding of MGP to HA is strongly dependent on the ionic environment, with free calcium ions increasing and inorganic phosphate and magnesium ions decreasing the binding affinity [79].

Two other proteins showed significantly different abundances on the two ceramics; antithrombin III was observed in higher amounts on TCP than on HA at 48 h, while cytochrome C was slightly more abundant on HA than on TCP after 168 h. Antithrombin III is known for its anticoagulant and antiinflammatory activity, and its suppression may lead to an uncontrolled cycle of coagulation and inflammation [80]. A previous study on titanium surfaces found adsorption of antithrombin III to be positively correlated with surface roughness, which also correlated with better osseointegration properties [81]. As mentioned before, the two ceramics differed not only in their chemical but also in their structural properties, which, as in the case of titanium, may have affected the antithrombin adsorption. Finally, cytochrome C, shown to play a role in mitochondrial electron transport and in apoptosis [82], has not been extensively discussed in relation to natural or synthetic minerals. A few studies exist in which cytochrome C was successfully coprecipitated with CaP on the surface of titanium and HA from supersaturated CaP solutions [83,84], but it is unclear whether the same mechanism played a role in this study and why it was more pronounced in HA than in TCP.

Taken together, the findings from the serum protein adsorption experiments highlighted the proteins that are highly abundant in the serum and/or have a high affinity to bind to CaP or to complex/coprecipitate calcium and/or phosphate ions. The extent of adsorption was suggested to be affected by the available surface for binding and by the ion exchange dynamics on the surface.

### 3.3 Protein production by hMSCs cultured on HA and TCP

Protein production by hMSCs cultured on HA and TCP for 8, 48, and 168 h was measured using LC-MS/MS. In total, 2049 proteins were identified with high confidence (FDR <0.01). Proteins were considered upregulated or downregulated if at least a 1.3-fold change (log_2_ >0.38) was detected with a p-value less than 0.05.Three hundred ninety three proteins were found to be more abundant in cells cultured on HA than in cells cultured on TCP after 8 h, while 238 and 222 proteins were identified as more abundant in cells cultured on HA than on TCP after 48 and 168 h, respectively. After 8, 48, and 168 h, 100, 107, and 72 proteins, respectively, were more abundant in cells cultured on TCP than on HA (Fig. 1c and d, Tables S7–12).

**Fig. 2:**
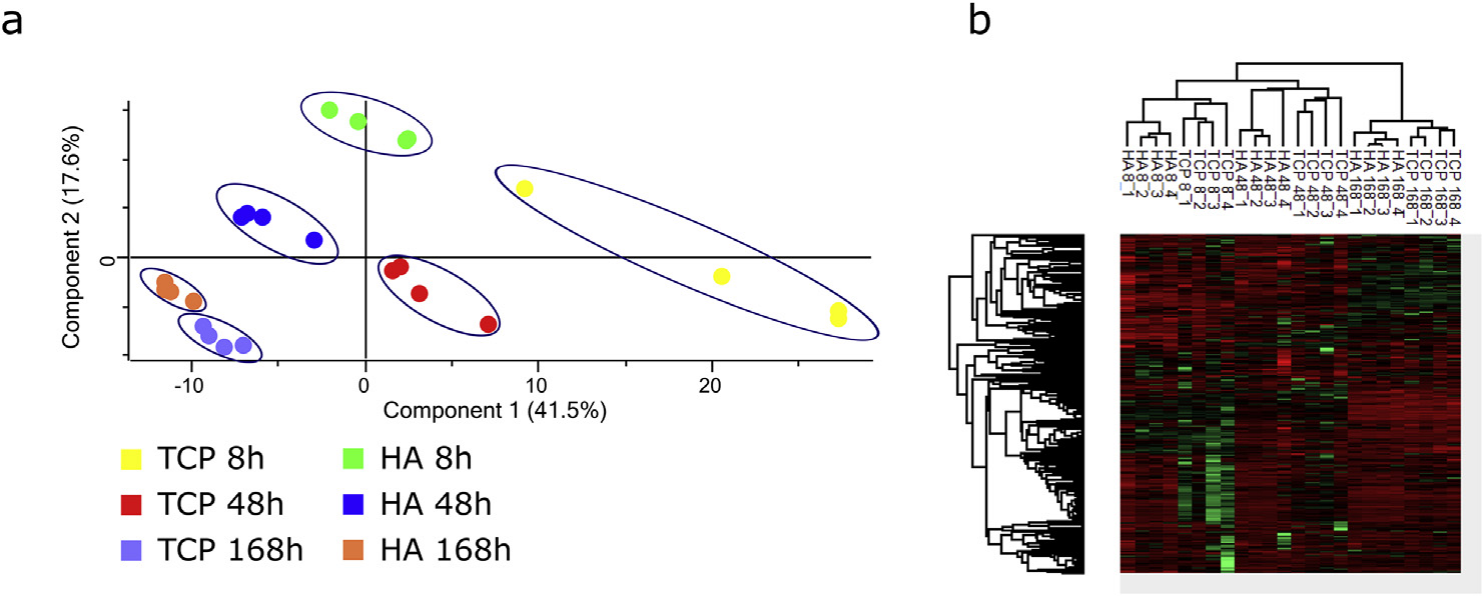
(a) Principal component analysis (PCA) of protein expression profiles in hMSCs cultured on HA and TCP for 8h, 48h, and 168h. Each dot represents a biological replicate. Samples are clustered based on similarity of their protein expression profiles based on the first two principal components (PC 1, representing 41.5% of explained variance, and PC 2, representing 17.6% of explained variance). There is a clear separation between protein profiles identified on different ceramics at all 3 time points. Blue ellipses have been manually added to indicate clusters. PCA projections are given in Table S13. (b) Heat map of protein expression in hMSCs cultured on HA and TCP for 8h, 4h, and 168h, generated using Perseus. Data were autoscaled (z-transformation), and Pearson correlation was used. Each column represents a biological replicate, and green and red represent upregulated and downregulated proteins, respectively. Tree diagram at top represents clustering of conditions, diagram at left represents clustering of proteins. (For interpretation of the references to colour in this figure legend, the reader is referred to the Web version of this article.) TCP, β-tricalcium phosphate; HA, hydroxyapatite; hMSCs, human mesenchymal stromal cells.

To identify potential patterns in these differences, PCA was performed based on the abundances of all 2049 proteins. The PCA plot (Fig. 2a) is based on the first and second principal components, covering 41.5% and 17.6% of the data set, respectively, and illustrates clustering of proteins by ceramic type and time point. The results show three main clusters, corresponding to the three time points, with each of these clusters further divided into two subclusters corresponding to HA and TCP. This division is in the same direction at each time point, and the changes over time occur in the same direction for both materials – indicating that the differences in protein expression profile between the two materials remain the same over time, while similar changes in protein expression profile over time are seen on both materials. Moreover, a decrease in the differences between HA and TCP, as well as in differences between the replicates of the same material, is seen over time, with the differences after 168 h being considerably smaller than those at the earlier time points. A heat map generated from this data set (Fig. 2b) confirms that the protein expression data are clustered by time point, and within each time point, the differences between cells cultured on the different ceramics can be observed.

Taken together, these results show that there is indeed a difference in protein expression between cells cultured on HA and those cultured on TCP. There is also a change in protein profile over time for cells cultured on both ceramics, and the differences between the ceramics are more pronounced at the earlier time points. These chronological differences can be explained by the fact that after 8 h, most cells are in direct contact with the ceramic surface. As cells proliferate, at later time points, an increasing proportion of cells are not in direct contact with the ceramic surface but instead are adhering to other cells in the pores of the ceramic. In an earlier work, we have used laser capture microdissection to show that the cells in direct contact with the ceramic surface form a distinct subpopulation with a different protein profile [28].

Based on this, we hypothesize that the differential production of proteins on the two ceramics is due to differences in their surface properties and to consequent differences in interfacial events such as ion exchange between the ceramic and the cell culture medium, already mentioned to affect binding of serum proteins to the ceramic surface above. The importance of the ceramic surface to cell behavior *in vitro* and osteoinduction *in vivo* has been suggested before. For example, in a comparative study culturing hMSCs and goat MSCs on HA and TCP ceramics and subsequent implantation in immunodeficient mice, distinct differences between the two ceramic surfaces were observed both in the amount of ectopic bone formation and in cell morphology *in vitro*. On TCP *in vitro*, the cells were largely interconnected and aligned, while on the HA ceramic, they showed a disorganized morphology with fewer connections. The authors suggested that the spatial distribution of the hMSCs was important in the determination of cell fate and affected extracellular matrix production [85]. In another study, different BCP ceramics containing both HA and TCP were compared in an *in vivo* dog intramuscular model. The authors reported that microstructural properties of the surface, rather than macrostructure or surface chemistry, were determinative in promoting osteoclastogenesis and triggering ectopic bone formation [14]. Similarly, Barradas et al. [54] observed that ceramic microstructure determined cell attachment and spreading and suggested that this initial response to the materials affects the extent of ectopic bone formation induced by the materials. We have also demonstrated that when hMSCs are cultured on TCP, only the cells in direct contact with the ceramic surface produce PC-1 [28], a protein that has been shown to play an important role in ectopic and orthotopic bone formation [86]. Taken together, these studies show that the surface properties of the different ceramics play an important role in the protein-level differences we have observed.

One of the proteins that was found to be more expressed in cells cultured on TCP than on HA at all time points was OPN, which is involved in matrix mineralization processes and is a well-known marker of osteogenic differentiation [87]. In addition to the gene-level analysis detailed in a previous study [28], we performed an immunohistochemical analysis which confirmed the production of OPN by cells cultured on TCP but not by those cultured on HA. The results of this analysis are detailed in the Supplementary Information. It was noted that on TCP, OPN was observed in direct contact with the surface of the material. This phenomenon resembles the biphasic pattern of OPN expression observed during intramembranous bone formation [88], which is the same route by which CaP-induced ectopic bone formation occurs [10]. This biphasic pattern has been attributed to the multiple functions of OPN during the osteogenesis process. On the one hand, OPN expression is essential for activating several signaling cascades that are important for cell survival and proliferation and for matrix mineralization, while on the other hand, OPN overexpression may inhibit HA crystallization and mineral deposition [88,89].

### 3.4 Pathway analysis of proteins identified in cells cultured on HA and TCP

**Fig. 3:**
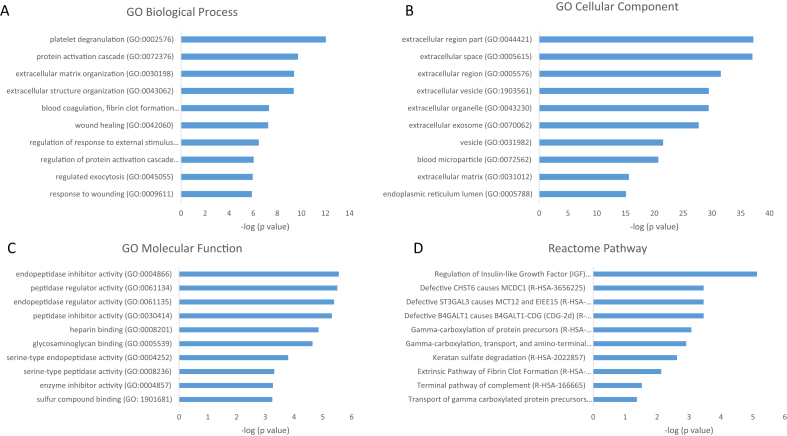
Top 10 GO terms with respect to (A) biological process, (B) cellular component, (C) molecular function, and (D) Reactome pathway of proteins significantly upregulated on TCP after 8 h, together with –log_10_ of p-values. Exact values are given in Table S14. TCP, β-tricalcium phosphate; GO, Gene Ontology.

**Fig. 4:**
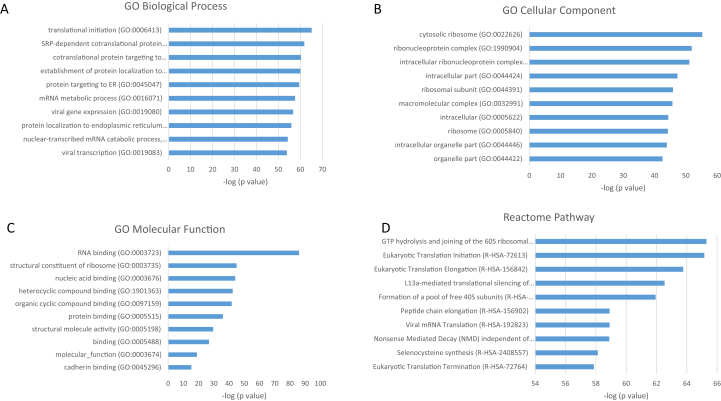
Top 10 GO terms with respect to (A) biological process, (B) cellular component, (C) molecular function, and (D) Reactome pathway of proteins significantly upregulated on HA after 8 h, together with –log_10_ of p-values. Exact values are given in Table S15. HA, hydroxyapatite; GO, Gene Ontology.

To obtain deeper understanding of the biological relevance of the aforementioned results, they were analyzed based on systematic GO Consortium annotations for biological process, cellular components, and molecular functions. The GO terms were ranked based on ascending overrepresentation and p-value corrected for multiple testing. Fig. 3, Fig. 4 show the top 10 most significant terms for each GO category, along with the top 10 most enriched pathways, after 8-h hMSC culture.

The proteins that were upregulated on TCP compared with HA after 8 h were found to be enriched for, among others, platelet degranulation, extracellular matrix organization, and wound healing. Most of these proteins were shown to be related to the extracellular region, while the analysis of the molecular function indicated enrichment of peptidase activity and binding to components of the extracellular matrix. Proteins that were upregulated on HA compared with TCP after 8 h were associated with protein translation and localization to the extracellular region and membrane. These proteins were likely to be spatially associated with the ribosome, while the molecular function analysis revealed enrichment for binding to mRNA, rRNA, and proteins, as well as binding to cell adhesion structure. The pathway analyses after 8 h, carried out based on the Reactome Pathway Database, indicated that the upregulated proteins on TCP were most enriched in pathways corresponding to platelet activation, formation of fibrin clot, insulin-like growth factor, lipoproteins, and gamma-carboxylated protein, whereas the proteins upregulated on HA were predominantly related to eukaryotic translation activity. This protein production may correspond to the known early stages of bone healing, in which a blood clot forms at the defect site, resulting in the triggering of an inflammatory response [90]. It follows that if blood clotting-related proteins are selectively produced on the surface of TCP but not HA, osteoinduction may be mediated by production of these proteins in the hours immediately after implantation leading to a similar inflammatory response.

**Fig. 5:**
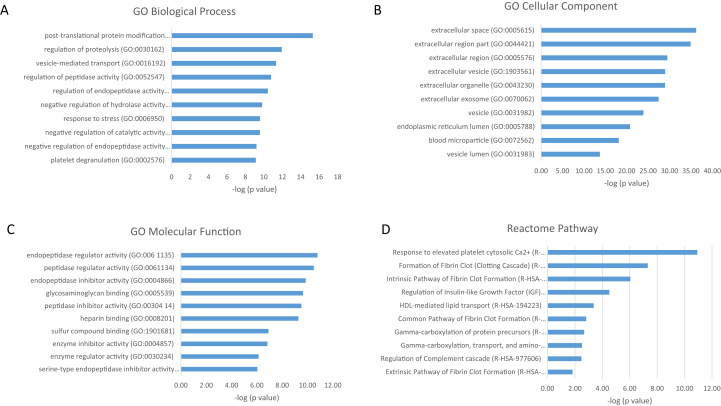
Top 10 GO terms with respect to (A) biological process, (B) cellular component, (C) molecular function, and (D) Reactome pathway of proteins significantly upregulated on TCP after 48 h, together with –log_10_ of p-values. Exact values are given in Table S16. TCP, β-tricalcium phosphate; GO, Gene Ontology.

**Fig. 6:**
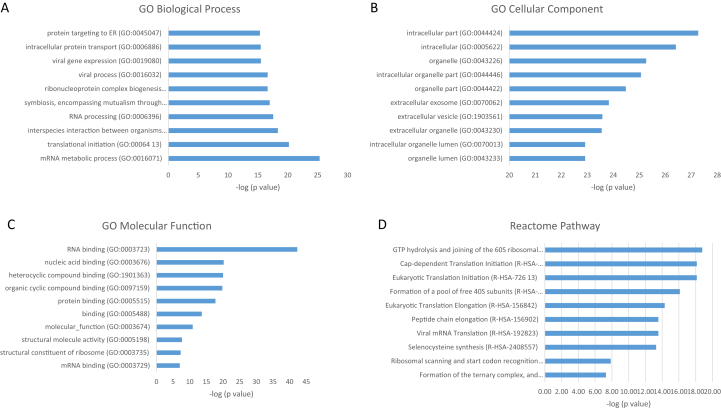
Top 10 GO terms with respect to (A) biological process, (B) cellular component, (C) molecular function, and (D) Reactome pathway of proteins significantly upregulated on HA after 48 h, together with –log_10_ of p-values. Exact values are given in Table S17. HA, hydroxyapatite; GO, Gene Ontology.

After 48 h, the proteins upregulated on TCP corresponded, among others, to posttranslational protein modifications, regulation of proteolysis, and vesicle-mediated transport, whereas proteins upregulated on HA were related to mRNA metabolic processes, translational initiation, and RNA processing. Most upregulated proteins on TCP were associated with the extracellular space, while the upregulated proteins on HA were associated with the intracellular regions. Pathway analysis indicated that proteins enriched on TCP corresponded to the extrinsic pathway of fibrin clot formation, gamma-carboxylation, transport, and amino-terminal cleavage of proteins, whereas proteins enriched on HA showed pathways involved in the formation of the ribosomal complex (Fig. 5, Fig. 6). From these results, the increase in proteins and processes related to transport and extracellular regions implies that TCP stimulates processes related to extracellular matrix production, which gene-level studies have suggested is important in the process of osteoinduction [21].

**Fig. 7:**
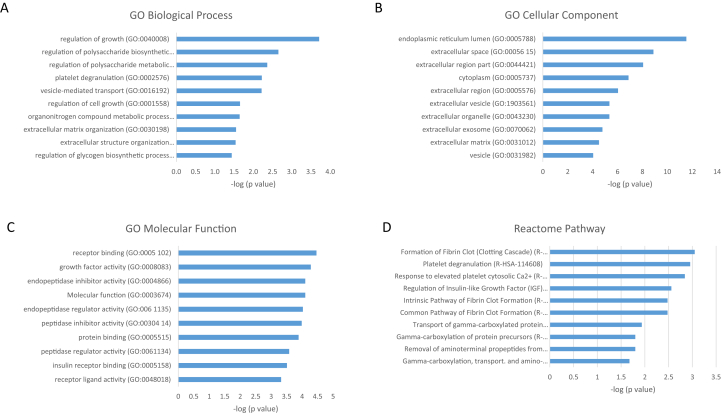
Top 10 GO terms with respect to (A) biological process, (B) cellular component, (C) molecular function, and (D) Reactome pathway of proteins significantly upregulated on TCP after 168 h, together with –log_10_ of p-values. Exact values are given in Table S18. TCP, β-tricalcium phosphate; GO, Gene Ontology.

**Fig. 8:**
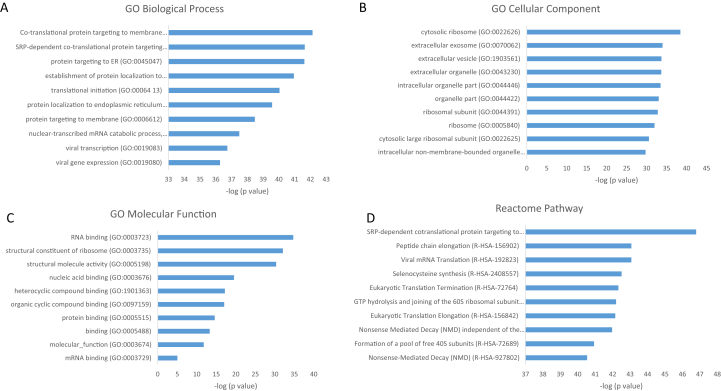
Top 10 GO terms with respect to (A) biological process, (B) cellular component, (C) molecular function, and (D) Reactome pathway of proteins significantly upregulated on HA after 168 h, together with –log_10_ of p-values. Exact values are given in Table S19. HA, hydroxyapatite; GO, Gene Ontology.

Finally, for the proteins upregulated on TCP after 168 h, associations with biological processes related to platelet activation and extracellular matrix production were also found. While processes related to wound healing and clot formation were not detected anymore, new terms related to regulation of growth and to polysaccharide processing appeared. Cellular components were similar to the other time points. For molecular functions, in addition to peptidase activity, terms related to growth factor activity and receptor binding were determined, possibly indicating the beginning of differentiation. The pathway analysis showed similar results to those obtained for other time points, with the fibrin clot formation pathway still being present, whereas the lipoprotein processing pathway was not detected. The analyses of the cellular components and molecular functions reflected regulatory and inhibitory activity of peptidase and endopeptidase as part of extracellular region activity for the proteins upregulated on TCP versus HA (Fig. 7, Fig. 8). It should be noted that at this late time point, the number of proteins that were significantly upregulated on TCP compared with HA was considerably lower than at the earlier time points. This leads to lower numbers of mapped changed proteins which may result in GO terms not reaching significance. For the proteins upregulated on HA, the analysis of biological processes, molecular functions, and pathways showed classes of processes similar to those at the earlier time points. For the cellular components, in addition to ribosomal locations, at this late time point, the extracellular space appears to play a more prominent role. The appearance of terms related to intracellular space may indicate changes in organelle activities, such as a switch in mitochondrial activities, which are known to occur during differentiation of MSCs. For example, it has been shown that the metabolic pattern of MSCs is more dependent on glycolysis, while in the MSC-derived differentiated cells, a switch to oxidative phosphorylation along with increased mitochondrial biogenesis is observed. Consequently, dynamic changes in metabolic activity of the cells are expected during osteogenic differentiation [91]. Therefore, the highlighted intracellular activities in cellular component terms at 8h and 48h for hMSCs cultured on HA may indicate the onset of differentiation. This phase may have taken place in the cells cultured on TCP at even earlier time points. The appearance of extracellular space in the cellular components of proteins upregulated on HA after 168h may suggest a later, less pronounced effect of HA on extracellular matrix production compared with TCP.

Taken together, based on the GO analysis performed on differentially produced proteins by cells cultured on HA and TCP at the three time points, it can be concluded that distinct biological activities were predominantly determined by the ceramic type. Within the tested period of time of 168 h, the cell culture time seemed to play a less important role, particularly in HA, which may be an indication that the initiation of certain processes, such as matrix production, is delayed in HA compared with TCP. Overall, the proteins that were found to be more abundant on the osteoinductive TCP than on the non-osteoinductive HA were involved in processes related to wound healing, cell proliferation, and extracellular matrix production. On the other hand, the proteins showing a higher abundance on HA than on TCP were more involved in processes related to protein production, translation, localization, and secretion. It should be noted that ribosomal proteins, which are also upregulated on HA, have been linked to cartilage formation and the differentiation of hMSCs to chondrocytes [92], while the process of CaP ceramics–induced ectopic bone formation is intramembranous, thus not occurring via a cartilaginous intermediate [10].

As with the discussion of serum protein adsorption, it is difficult to draw firm conclusions on the biological activities of cells cultured on TCP and HA from these data, as the materials differ in multiple respects and some confounding effects may be present. However, as with serum proteins, it can be speculated that events at the ceramic surface play an important role. For instance, we have shown in a previous study that the amount of calcium and inorganic phosphate ions in cell culture medium differs depending on whether the cells are cultured on HA or TCP, with significantly lower levels of these ions being detected in the medium of cells cultured on TCP [28]. This suggests that more of these ions from the medium are adsorbed or otherwise deposited on the ceramic surface, potentially resulting in a localized depletion of the ions close to the surface which has been suggested as a key step in osteoinduction [11]. However, further investigation, using model materials that allow decoupling of individual properties, is required to render this more than a speculative explanation of our observations.

### 3.5 Protein network visualizations

To further investigate the interactions between proteins involved in osteoinduction and their dependence on material properties, a new series of hMSC culture experiments was performed on the non-osteoinductive HA and BCP1300 and osteoinductive TCP and BCP1150, followed by an LC-MS/MS analysis. For this analysis, a set of ‘top 50’ proteins (data set 1 - Table S20) that were those previously determined to be most significantly enriched (lowest p-values in an ANOVA test, n = 3) after 168 h in hMSCs cultured on osteoinductive TCP and BCP1150 compared with those cultured on non-osteoinductive HA and BCP1300 [28] was used. It should be noted that most of these proteins were overexpressed on all CaP ceramics, both osteoinductive and non-osteoinductive ceramics, compared with cells cultured on 2D tissue culture polystyrene plates. This suggests that they are upregulated either in a 3D environment or in response to the presence of non-specific CaPs.

From this data set, 31 interactions were found after filtering using the combined interaction score generated using the STRING database [44] based on a range of text and database evidence channels as detailed in Section 2.7. These formed two significant clusters and a set of other individual significant interactions.

**Fig. 9:**
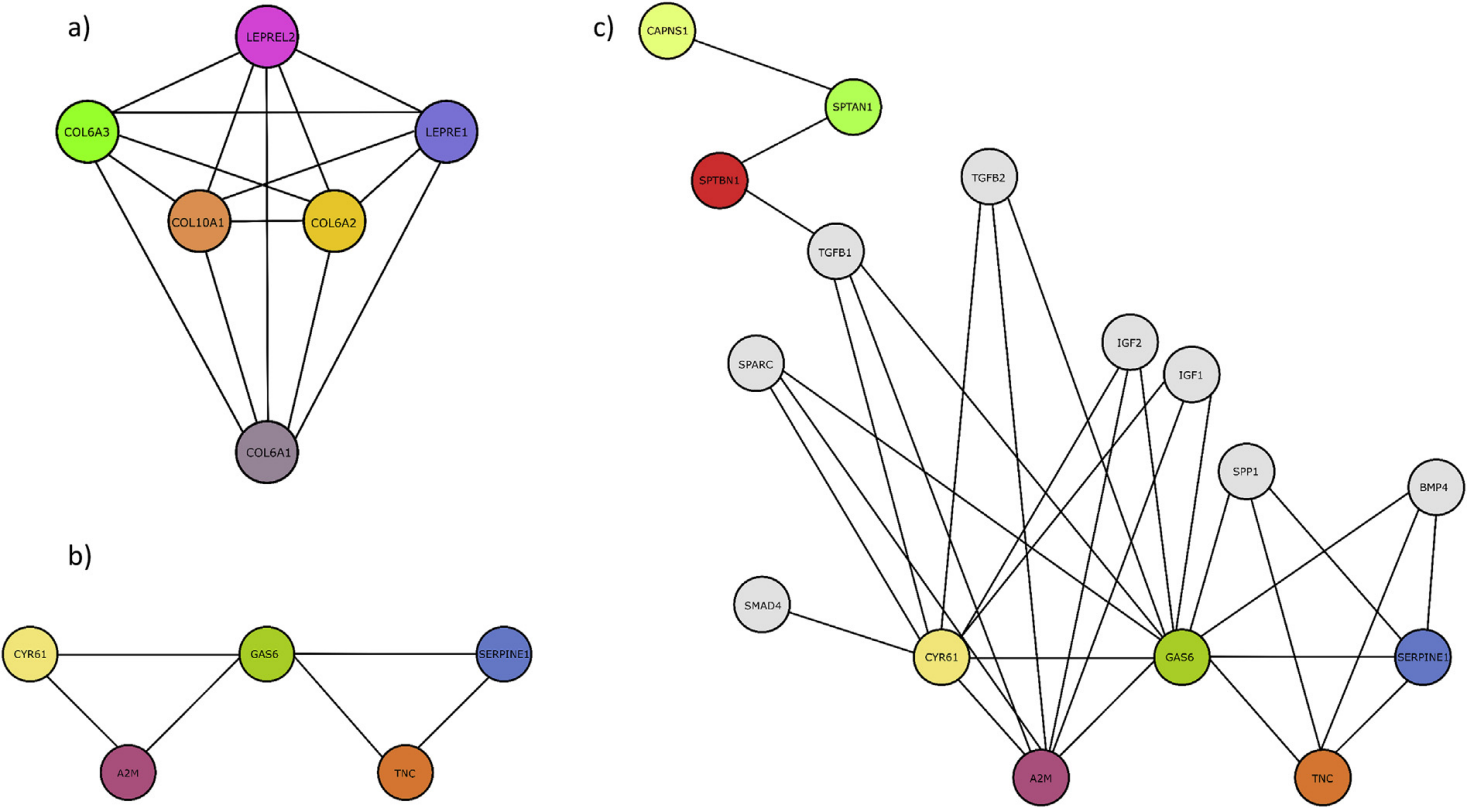
Visualization of protein networks found by cluster analysis. Proteins in data set 1 are colored for ease of distinction and those in data set 2 are gray. Lines between nodes represent interaction. (A) Largest cluster from data set 1. (B) Second cluster from data set 1. (C) Interactions of cluster B with proteins involved in BMP signaling cascade.

The largest cluster (Fig. 9a) included collagen VI subunits A1, A2, and A3, collagen X subunit A1, prolyl-3 hydroxylase 1, and prolyl-3 hydroxylase 3. The p-value of this cluster was 0.017, with strong interactions found between all of its components. These proteins either are subunits of collagen or are involved in collagen cross-linking [93,94], and thus potentially relevant to the process of osteogenesis clear as collagen production is a main step in the formation of the matrix which mineralizes to form bone [90]. This supports the hypothesis that the upregulation of proteins regulated to extracellular regions on TCP found by the GO analysis (Section 3.4) corresponds to increased levels of matrix formation.

A second cluster from data set 1 consisted of growth arrest-specific 6 (GAS6), cysteine-rich angiogenic inducer 61 (CYR61), TNC, alpha-2-macroglobulin (A2M), and plasminogen activator inhibitor 1 (PAI1 or SERPINE1) (Fig. 9b). The p-value for this cluster was 0.05; all four other proteins exhibited strong interactions with GAS6, but no interaction was found between PAI1 or A2M and TNC, and the interaction between PAI1 and CYR61 was not strong enough to be included, with a score of 0.563 compared with the threshold of 0.9. In addition, no literature evidence for the interaction of A2M and PAI1 was found; the relatively high score of this interaction is largely due to gene expression data. The most interesting interaction from this cluster is between GAS6 and CYR61, which has already been extensively studied. GAS6 produced by osteoblasts stimulates osteoclastic function by acting as a ligand to the tyro 3 receptor tyrosine kinase [95]. Experiments in melanoma cells also showed that GAS6 downregulates expression of CYR61. CYR61, in turn, increases proliferation and osteoblastic differentiation in a BMP2-dependent manner through the α_v_β_3_ integrin/integrin-linked kinase/ extracellular signal-regulated kinase (ERK) pathway [96]. These two interactions suggest a mechanism for the involvement of GAS6 in the regulation of osteogenesis.

Finally, a range of interactions between groups of proteins each of which only had one or two interactions, therefore not qualifying as a cluster, were found from data set 1. Of these, the most interesting is the interaction between cofilin 1 (CFL1) and actin B. CFL1 can (de-)polymerize both F- and G-actin, and the presence of intranuclear actin has been shown to induce expression of osteogenic genes such as OSX and OC [97]. The transfer of G-actin into the nucleus is CFL1 dependent, so CFL1 appears also to be linked to osteogenic signaling.

The addition of the 39 further proteins known to be linked to the BMP and Wnt signaling pathways (Table S21) to the set of 50 found by the LC-MS/MS analysis gave a second, larger data set referred to as data set 2. Most new interactions found in this expanded data set were between the additional 39 proteins,as these proteins are already known to be involved in well-characterized signaling pathways; these interactions will not be discussed further. No interactions were found between the members of the first, collagen-related cluster and the 39 BMP- and Wnt-related proteins.

The proteins in the second cluster from data set 1 were found to interact with growth factors largely involved in the BMP signaling cascade (Fig. 9c). In particular, PAI1 was found to interact with IGF1, IGF2, TGFβ-2, SPARC, and SMAD4 [98]. However, yet little is known about the way in which these interactions influence osteogenesis.

A range of new unclustered interactions were also found. Of these, the most interesting are the interactions of ectonucleotide pyrophosphatase/phosphodiesterase (ENPP1) with alkaline phosphatase non-specific isozyme (ALPL) and integrin-binding sialoprotein. The role of ENPP1 in osteoinduction has been investigated in previous work in our laboratory [28], and both ENPP1 and ALPL are regulators of bone mineralization and involved in gene polymorphisms affecting bone size [99].

In conclusion, we have found that some of the proteins differentially expressed by hMSCs on osteoinductive versus non-osteoinductive ceramic surfaces participate in pathways related to already known signaling pathways. These can be in part linked to the results of the GO analysis showing enrichment of proteins related to collagen production and growth factor signaling.

## 4 Conclusions

With the aims of adding to the existing knowledge of osteoinductive biomaterials and obtaining a deeper insight into the mechanisms governing the process of osteoinduction using proteomics, we were able to analyze the adsorption of serum proteins on two CaP ceramics with well-defined properties and osteoinductive potential. A larger number of proteins were identified to be adsorbed on an osteoinductive TCP than on a non-osteoinductive HA, and an increase in the number of proteins adsorbed specifically on TCP was seen over time, plausibly due to a larger surface area of TCP. Of the four proteins that were present on both ceramics but in significantly different amounts, two are known for their affinity to bind to CaPs and to be related to regulation of ectopic and orthotopic calcification and bone formation.

In addition, we monitored and compared protein profiles of hMSCs cultured on these ceramics. The protein profiles of hMSCs cultured on HA and TCP were significantly different and could be separated in a PCA plot both in terms of material type and cell culture time. Over time, the protein profiles of hMSCs cultured on these two ceramics became more similar to each other. A key difference between the two ceramics was that the proteins identified as upregulated in hMSCs cultured on TCP were related to wound healing, cell proliferation, and extracellular matrix production, while those seen to be upregulated in hMSCs cultured on HA were related to protein production, translation, localization, and secretion.

Finally, we were able to use cluster analysis on the results of a broader experiment to find links between the proteins which we found to be upregulated on osteoinductive ceramics and those involved in pathways known to be related to osteogenesis.

It should be noted that, similar to most *in vitro* systems, the one we used here does not fully recapitulate the *in vivo* situation upon implantation of a CaP ceramic in the body. The initial protein adsorption occurring in the environment of the body is expected to be different from that in a cell culture medium containing serum, influenced, for example, by a slightly altered pH of the environment upon implantation, slightly hypoxic conditions, different amounts of calcium and inorganic phosphate, presence of other cytokines, and so on. Moreover, hMSCs will not be the only and definitely not the first cells that attach to the ceramic surface in the body, resulting in a different situation than described here. One could even argue that the conditions tested here are more representative of a tissue engineered construct in which the ceramic is used as a carrier and hMSCs are added to it before implantation. Therefore, the results obtained here cannot be directly extrapolated to the *in vivo* situation because they represent only few important aspects of the complex process of osteoinduction.

Therefore, further investigation is needed to fully determine the biological relevance of the differential adsorption of serum proteins on HA and TCP surfaces and the different protein expression profiles of hMSCs cultured on osteoinductive and non-osteoinductive ceramics. These future studies should ideally include hMSCs from multiple donors and an analysis of the behavior of subpopulations of hMSCs on the different ceramics. MSCs, characterized as multipotent stem cells, comprise heterogeneous population of progenitor cells. Therefore, in addition to donor dependency, their osteogenic differentiation potential may also vary among different subpopulations [100].

Within the limitations of this study, we have shown that a protein profiling approach as applied here can be a useful tool to investigate protein adhesion to biomaterials and the effect of biomaterials on protein production, using clinically relevant biological systems. This type of experiment has the potential to contribute to our understanding of the mechanisms underlying the bioactivity of biomaterials and the effect of material properties on these mechanisms.

## Credit author statement

**Ziryan Othman:** Conceptualization, Methodology, Investigation, Validation, Writing - original draft. **Ronny J.C. Mohren:** Formal analysis, Investigation, Resources, Writing - original draft, Writing - review & editing, Visualization. **Berta Cillero Pastor:** Formal analysis, Investigation, Resources, Writing - original draft, Writing - review & editing. **Zhaoji Shen:** Formal analysis, Methodology, Visualization, Writing - original draft **Yves S.N.W. Lacroix:** Formal analysis, Methodology, Visualization, Writing - original draft. **Alexander P.M. Guttenplan:** Formal analysis, Methodology, Visualization, Writing - original draft, Writing - review & editing. **Zeinab Tahmasebi Birgani:** Formal analysis, Methodology, Visualization, Writing - review & editing. **Lars Eijssen:** Formal analysis, Methodology, Visualization, Writing - original draft. **Theo M. Luider:** Conceptualization, Methodology, Investigation, Writing - original draft. **Sabine van Rijt:** Writing - original draft, Writing - review & editing. **Pamela Habibovic**: Conceptualization, Methodology, Resources, Writing - review & editing, Supervision, Project administration, Funding acquisition

## Data Availability

The raw data required to reproduce these findings cannot be shared at this time due to technical limitations. The processed data required to reproduce these findings are available from https://hdl.handle.net/10411/TKX78B.

## Declaration of competing interest

The authors declare no conflict of interest.

## References

[1] Holzapfel B.M., Reichert J.C., Schantz J.T., Gbureck U., Rackwitz L., Nöth U., Jakob F., Rudert M., Groll J., Hutmacher D.W. (2013). How smart do biomaterials need to be? A translational science and clinical point of view. Adv. Drug Deliv. Rev..

[2] Best S.M., Porter A.E., Thian E.S., Huang J. (2008). Bioceramics: past, present and for the future. J. Eur. Ceram. Soc..

[3] Haugen H.J., Lyngstadaas S.P., Rossi F., Perale G. (2019). Bone grafts: which is the ideal biomaterial?. J. Clin. Periodontol..

[4] Zhang M., Matinlinna J.P., Tsoi J.K.H., Liu W., Cui X., Lu W.W., Pan H. (2019). Recent developments in biomaterials for long-bone segmental defect reconstruction: a narrative overview. J. Orthop. Transl.

[5] Liu B., xing Lun D. (2012). Current application of β-tricalcium phosphate composites in orthopaedics. Orthop. Surg..

[6] Albrektsson T., Johansson C. (2001). Osteoinduction, osteoconduction and osseointegration. Eur. Spine J..

[7] Habraken W., Habibovic P., Epple M., Bohner M. (2016). Calcium phosphates in biomedical applications: materials for the future?. Mater. Today.

[8] Jodati H., Yılmaz B., Evis Z. (2020). A review of bioceramic porous scaffolds for hard tissue applications: effects of structural features. Ceram. Int..

[9] Martin V., Bettencourt A. (2018). Bone regeneration: biomaterials as local delivery systems with improved osteoinductive properties. Mater. Sci. Eng. C.

[10] Barradas A.M.C., Yuan H., van Blitterswijk C.A., Habibovic P. (2011). Osteoinductive biomaterials: current knowledge of properties, experimental models and biological mechanisms. Eur. Cell. Mater..

[11] Bohner M., Miron R.J. (2019). A proposed mechanism for material-induced heterotopic ossification. Mater. Today.

[12] Barba A., Diez-Escudero A., Espanol M., Bonany M., Sadowska J.M., Guillem-Marti J., Öhman-Mägi C., Persson C., Manzanares M.C., Franch J., Ginebra M.P. (2019). Impact of biomimicry in the design of osteoinductive bone substitutes: nanoscale matters. ACS Appl. Mater. Interfaces.

[13] Barba A., Diez-Escudero A., Maazouz Y., Rappe K., Espanol M., Montufar E.B., Bonany M., Sadowska J.M., Guillem-Marti J., Öhman-Mägi C., Persson C., Manzanares M.C., Franch J., Ginebra M.P. (2017). Osteoinduction by foamed and 3D-printed calcium phosphate scaffolds: effect of nanostructure and pore architecture. ACS Appl. Mater. Interfaces.

[14] Davison N.L., Su J., Yuan H., van den Beucken J.J.J.P., De Bruijn J.D., Barrere-de Groot F. (2015). Influence of surface microstructure and chemistry on osteoinduction and osteoclastogenesis by biphasic calcium phosphate discs. Eur. Cell. Mater..

[15] Davison N.L., Luo X., Schoenmaker T., Everts V., Yuan H., Barrère-de Groot F., de Bruijn J.D. (2014). Submicron-scale surface architecture of tricalcium phosphate directs osteogenesis in vitro and in vivo. Eur. Cell. Mater..

[16] Barba A., Maazouz Y., Diez-Escudero A., Rappe K., Espanol M., Montufar E.B., Öhman-Mägi C., Persson C., Fontecha P., Manzanares M.C., Franch J., Ginebra M.P. (2018). Osteogenesis by foamed and 3D-printed nanostructured calcium phosphate scaffolds: effect of pore architecture. Acta Biomater..

[17] Ripamonti U., Duarte R., Ferretti C. (2014). Re-evaluating the induction of bone formation in primates. Biomaterials.

[18] Dobbenga S., Fratila-Apachitei L.E., Zadpoor A.A. (2016). Nanopattern-induced osteogenic differentiation of stem cells – a systematic review. Acta Biomater..

[19] Groen N., Tahmasebi N., Shimizu F., Sano Y., Kanda T., Barbieri D., Yuan H., Habibovic P., van Blitterswijk C.A., de Boer J. (2015). Exploring the material-induced transcriptional landscape of osteoblasts on bone graft materials. Adv. Healthc. Mater..

[20] Barradas A.M.C., Monticone V., Hulsman M., Danoux C., Fernandes H., Tahmasebi Birgani Z., Barrère-De Groot F., Yuan H., Reinders M., Habibovic P., Van Blitterswijk C., De Boer J. (2013). Molecular mechanisms of biomaterial-driven osteogenic differentiation in human mesenchymal stromal cells, Integr. Biol. (United Kingdom).

[21] Groen N., Yuan H., Hebels D.G.A.J., Koçer G., Mbuyi F., LaPointe V., Truckenmüller R., van Blitterswijk C.A., Habibović P., de Boer J. (2017). Linking the transcriptional landscape of bone induction to biomaterial design parameters. Adv. Mater..

[22] Tang Z., Tan Y., Ni Y., Wang J., Zhu X., Fan Y., Chen X., Yang X., Zhang X. (2017). Comparison of ectopic bone formation process induced by four calcium phosphate ceramics in mice. Mater. Sci. Eng. C.

[23] Davison N.L., ten Harkel B., Schoenmaker T., Luo X., Yuan H., Everts V., Barrère-de Groot F., de Bruijn J.D. (2014). Osteoclast resorption of beta-tricalcium phosphate controlled by surface architecture. Biomaterials.

[24] Othman Z., Cillero Pastor B., van Rijt S., Habibovic P. (2018). Understanding interactions between biomaterials and biological systems using proteomics. Biomaterials.

[25] Yang M.H., Yuan S.S., Chung T.W., Bin Jong S., Lu C.Y., Tsai W.C., Chen W.C., Lin P.C., Chiang P.W., Tyan Y.C. (2014). Characterization of silk fibroin modified surface: a proteomic view of cellular response proteins induced by biomaterials. BioMed Res. Int..

[26] Yuan H., Fernandes H., Habibovic P., de Boer J., Barradas A.M.C., de Ruiter A., Walsh W.R., van Blitterswijk C.A., de Bruijn J.D. (2010). Osteoinductive ceramics as a synthetic alternative to autologous bone grafting. Proc. Natl. Acad. Sci. Unit. States Am..

[27] Habibovic P., Yuan H., Van Den Doel M., Sees T.M., Van Blitterswijk C.A., De Groot K. (2006). Relevance of osteoinductive biomaterials in critical-sized orthotopic defect. J. Orthop. Res..

[28] Othman Z., Fernandes H., Groot A.J., Luider T.M., Alcinesio A., de Melo Pereira D., Guttenplan A.P.M., Yuan H., Habibovic P. (2019). The role of ENPP1/PC-1 in osteoinduction by calcium phosphate ceramics. Biomaterials.

[29] Li S., de Wijn J.R., Li J., Layrolle P., de Groot K. (2003). Macroporous biphasic calcium phosphate scaffold with high permeability/porosity ratio. Tissue Eng..

[30] Yuan H., Van Den Doel M., Li S., Van Blitterswijk C.A. (2002). A comparison of the osteoinductive potential of two calcium phosphate ceramics implanted intramuscularly in goats. J. Mater. Sci. Mater. Med..

[31] Mentink A., Hulsman M., Groen N., Licht R., Dechering K.J., van der Stok J., Alves H.A., Dhert W.J., van Someren E.P., Reinders M.J.T., van Blitterswijk C.A., De Boer J. (2013). Predicting the therapeutic efficacy of MSC in bone tissue engineering using the molecular marker CADM1. Biomaterials.

[32] Stingl C., Van Vilsteren F.G.I., Guzel C., Kate F.J.W., Visser M., Kausilia K., Bergman J.J., Luider T.M. (2011). Reproducibility of protein identification of selected cell types in Barrett's esophagus analyzed by combining laser - capture micro - dissection and mass spectrometry. J. Proteome Res..

[33] Craig R., Beavis R.C. (2003). A method for reducing the time required to match protein sequences with tandem mass spectra. Rapid Commun. Mass Spectrom..

[34] Searle B.C., Turner M., Nesvizhskii A.I. (2008). Improving sensitivity by probabilistically combining results from multiple MS/MS search methodologies. J. Proteome Res..

[35] Keller A., Nesvizhskii A.I., Kolker E., Aebersold R. (2002). Empirical statistical model to estimate the accuracy of peptide identifications made by MS/MS and database search. Anal. Chem..

[36] Nesvizhskii A.I., Keller A., Kolker E., Aebersold R. (2003). A statistical model for identifying proteins by tandem mass spectrometry. Anal. Chem..

[37] Searle B.C. (2010). Scaffold: a bioinformatic tool for validating MS/MS-based proteomic studies. Proteomics.

[38] Tyanova S., Temu T., Cox J. (2016). The MaxQuant computational platform for mass spectrometry-based shotgun proteomics. Nat. Protoc..

[39] Tyanova S., Temu T., Sinitcyn P., Carlson A., Hein M.Y., Geiger T., Mann M., Cox J. (2016). The Perseus computational platform for comprehensive analysis of (prote)omics data. Nat. Methods.

[40] Mi H., Muruganujan A., Ebert D., Huang X., Thomas P.D. (2019). PANTHER version 14: more genomes, a new PANTHER GO-slim and improvements in enrichment analysis tools. Nucleic Acids Res..

[41] Mi H., Thomas P. (2009). PANTHER pathway: an ontology-based pathway database coupled with data analysis tools. Methods Mol. Biol..

[42] Gene Ontology Consortium (2015). Gene ontology Consortium: going forward. Nucleic Acids Res..

[43] Jassal B., Matthews L., Viteri G., Gong C., Lorente P., Fabregat A., Sidiropoulos K., Cook J., Gillespie M., Haw R., Loney F., May B., Milacic M., Rothfels K., Sevilla C., Shamovsky V., Shorser S., Varusai T., Weiser J., Wu G., Stein L., Hermjakob H., D'Eustachio P. (2020). The reactome pathway knowledgebase. Nucleic Acids Res..

[44] Szklarczyk D., Franceschini A., Wyder S., Forslund K., Heller D., Huerta-Cepas J., Simonovic M., Roth A., Santos A., Tsafou K.P., Kuhn M., Bork P., Jensen L.J., Von Mering C. (2015). STRING v10: protein-protein interaction networks, integrated over the tree of life. Nucleic Acids Res..

[45] Shannon P., Markiel A., Ozier O., Baliga N.S., Wang J.T., Ramage D., Amin N., Schwikowski B., Ideker T. (2003). Cytoscape: a software environment for integrated models of biomolecular interaction networks. Genome Res..

[46] Kanehisa M., Sato Y., Furumichi M., Morishima K., Tanabe M. (2019). New approach for understanding genome variations in KEGG. Nucleic Acids Res..

[47] Jensen L.J., Lagarde J., von Mering C., Bork P. (2004). ArrayProspector: a web resource of functional associations inferred from microarray expression data. Nucleic Acids Res..

[48] Lin G.L., Hankenson K.D. (2011). Integration of BMP, Wnt, and notch signaling pathways in osteoblast differentiation. J. Cell. Biochem..

[49] Dančík V., Seiler K.P., Young D.W., Schreiber S.L., Clemons P.A. (2010). Distinct biological network properties between the targets of natural products and disease genes. J. Am. Chem. Soc..

[50] Orlando B., Bragazzi N., Nicolini C. (2013). Bioinformatics and systems biology analysis of genes network involved in OLP (Oral Lichen Planus) pathogenesis. Arch. Oral Biol..

[51] Sbordone L., Sbordone C., Filice N., Menchini-Fabris G., Baldoni M., Toti P. (2009). Gene clustering analysis in human osseous remodeling. J. Periodontol..

[52] Nepusz T., Yu H., Paccanaro A. (2012). Detecting overlapping protein complexes in protein-protein interaction networks. Nat. Methods.

[53] Habibovic P., Kruyt M.C., Juhl M.V., Clyens S., Martinetti R., Dolcini L., Theilgaard N., Van Blitterswijk C.A. (2008). Comparative in vivo study of six hydroxyapatite-based bone graft substitutes. J. Orthop. Res..

[54] Barradas A.M.C., Yuan H., van der Stok J., Le Quang B., Fernandes H., Chaterjea A., Hogenes M.C.H., Shultz K., Donahue L.R., van Blitterswijk C., de Boer J. (2012). The influence of genetic factors on the osteoinductive potential of calcium phosphate ceramics in mice. Biomaterials.

[55] Ng A.M.H., Tan K.K., Phang M.Y., Aziyati O., Tan G.H., Isa M.R., Aminuddin B.S., Naseem M., Fauziah O., Ruszymah B.H.I. (2008). Differential osteogenic activity of osteoprogenitor cells on HA and TCP/HA scaffold of tissue engineered bone. J. Biomed. Mater. Res..

[56] Yamasaki H., Sakai H. (1992). Osteogenic response to porous hydroxyapatite ceramics under the skin of dogs. Biomaterials.

[57] Maazouz Y., Rentsch I., Lu B., Santoni B.L.G., Doebelin N., Bohner M. (2020). In vitro measurement of the chemical changes occurring within β-tricalcium phosphate bone graft substitutes. Acta Biomater..

[58] Welch J.H., Gutt W. (1961). 874. High-temperature studies of the system calcium oxide-phosphorus pentoxide. J. Chem. Soc..

[59] Nagai T., Miyake M., Maeda M. (2009). Thermodynamic measurement of calcium phosphates by double knudsen cell mass spectrometry. Metall. Mater. Trans. B Process Metall. Mater. Process. Sci..

[60] Gross K.A., Berndt C.C. (1998). Thermal processing of hydroxyapatite for coating production. J. Biomed. Mater. Res..

[61] Potgieter J.H., Potgieter S.S., Moja S.J., Mulaba-Bafubiandi A. (2002). An empirical study of factors influencing lime slaking. Part I: production and storage conditions. Miner. Eng..

[62] Rodriguez-Navarro C., Elert K., Ševčík R. (2016). Amorphous and crystalline calcium carbonate phases during carbonation of nanolimes: implications in heritage conservation. CrystEngComm.

[63] Lu H.B., Campbell C.T., Graham D.J., Ratner B.D. (2000). Surface characterization of hydroxyapatite and related calcium phosphates by XPS and TOF-SIMS. Anal. Chem..

[64] Samavedi S., Whittington A.R., Goldstein A.S. (2013). Calcium phosphate ceramics in bone tissue engineering: a review of properties and their influence on cell behavior. Acta Biomater..

[65] Rabe M., Verdes D., Seeger S. (2011). Understanding protein adsorption phenomena at solid surfaces. Adv. Colloid Interface Sci..

[66] Kaneko H., Kamiie J., Kawakami H., Anada T., Honda Y., Shiraishi N., Kamakura S., Terasaki T., Shimauchi H., Suzuki O. (2011). Proteome analysis of rat serum proteins adsorbed onto synthetic octacalcium phosphate crystals. Anal. Biochem..

[67] Zipfel P.F., Skerka C. (2009). Complement regulators and inhibitory proteins. Nat. Rev. Immunol..

[68] Baeriswyl V., Calzavarini S., Chen S., Zorzi A., Bologna L., Angelillo-Scherrer A., Heinis C. (2015). A synthetic factor XIIa inhibitor blocks selectively intrinsic coagulation initiation. ACS Chem. Biol..

[69] Bhan I. (2014). Vitamin D binding protein and bone health. Internet J. Endocrinol..

[70] Vargas S., Bouillon R., Van Baelen H., Raisz L.G. (1990). Effects of vitamin D-binding protein on bone resorption stimulated by 1,25 dihydroxyvitamin D3, Calcif. Tissue Int.

[71] Brylka L., Jahnen-Dechent W. (2013). The role of fetuin-A in physiological and pathological mineralization. Calcif. Tissue Int..

[72] Alberghina D., Giannetto C., Vazzana I., Ferrantelli V., Piccione G. (2011). Reference intervals for total protein concentration, serum protein fractions, and albumin/globulin ratios in clinically healthy dairy cows. J. Vet. Diagn. Invest..

[73] Seto J., Busse B., Gupta H.S., Schäfer C., Krauss S., Dunlop J.W.C., Masic A., Kerschnitzki M., Zaslansky P., Boesecke P., Catalá-Lehnen P., Schinke T., Fratzl P., Jahnen-Dechent W. (2012). Accelerated growth plate mineralization and foreshortened proximal limb bones in fetuin-A knockout mice. PloS One.

[74] Schinke T., Amendt C., Trindl A., Pöschke O., Müller-Esterl W., Jahnen-Dechent W. (1996). The serum protein α 2 -hs glycoprotein/fetuin inhibits apatite formation in vitro and in mineralizing calvaria cells. J. Biol. Chem..

[75] Szweras M., Liu D., Partridge E.A., Pawling J., Sukhu B., Clokie C., Jahnen-Dechent W., Tenenbaum H.C., Swallow C.J., Grynpas M.D., Dennis J.W. (2002). α2-HS glycoprotein/fetuin, a transforming growth factor β/bone morphogenetic protein antagonist, regulates postnatal bone growth and remodeling. J. Biol. Chem..

[76] Zebboudj A.F., Imura M., Boström K. (2002). Matrix GLA protein, a regulatory protein for bone morphogenetic protein-2. J. Biol. Chem..

[77] Dan H., Simsa-Maziel S., Reich A., Sela-Donenfeld D., Monsonego-Ornan E. (2012). The role of matrix Gla protein in ossification and recovery of the avian growth plate. Front. Endocrinol..

[78] Price P.A., Williamson M.K. (1985). Primary structure of bovine matrix Gla protein, a new vitamin K-dependent bone protein. J. Biol. Chem..

[79] Roy M.E., Nishimoto S.K. (2002). Matrix Gla protein binding to hydroxyapatite is dependent on the ionic environment: calcium enhances binding affinity but phosphate and magnesium decrease affinity. Bone.

[80] Levy J.H., Sniecinski R.M., Welsby I.J., Levi M. (2016). Antithrombin: anti-inflammatory properties and clinical applications, Thromb. Haemostasis.

[81] Romero-Gavilán F., Gomes N.C., Ródenas J., Sánchez A., Azkargorta M., Iloro I., Elortza F., García Arnáez I., Gurruchaga M., Goñi I., Suay J. (2017). Proteome analysis of human serum proteins adsorbed onto different titanium surfaces used in dental implants. Biofouling.

[82] Hüttemann M., Pecina P., Rainbolt M., Sanderson T.H., Kagan V.E., Samavati L., Doan J.W., Lee I. (2011). The multiple functions of cytochrome c and their regulation in life and death decisions of the mammalian cell: from respiration to apoptosis. Mitochondrion.

[83] Sogo Y., Ito A., Fukasawa K., Kondo N., Ishikawa Y., Ichinose N., Yamazaki A. (2005). Coprecipitation of cytochrome C with calcium phosphate on hydroxyapatite ceramic. Curr. Appl. Phys..

[84] Sogo Y., Ito A., Onoguchi M., Li X., Oyane A., Ichinose N. (2009). Formation of cytochrome C-apatite composite layer on NaOH- and heat-treated titanium. Mater. Sci. Eng. C.

[85] Prins H.-J., Fernandes H., Rozemuller H., van Blitterswijk C., de Boer J., Martens A.C.M. (2016). Spatial distribution and survival of human and goat mesenchymal stromal cells on hydroxyapatite and β -tricalcium phosphate. J. Tissue Eng. Regen. Med..

[86] Mackenzie N.C.W., Huesa C., Rutsch F., MacRae V.E. (2012). New insights into NPP1 function: lessons from clinical and animal studies. Bone.

[87] Weinreb M., Shinar D., Rodan G.A. (1990). Different pattern of alkaline phosphatase, osteopontin, and osteocalcin expression in developing rat bone visualized by in situ hybridization. J. Bone Miner. Res..

[88] Perrien D.S., Brown E.C., Aronson J., Skinner R.A., Montague D.C., Badger T.M., Lumpkin C.K. (2002). Immunohistochemical study of osteopontin expression during distraction osteogenesis in the rat. J. Histochem. Cytochem..

[89] Huang W., Carlsen B., Rudkin G., Berry M., Ishida K., Yamaguchi D.T., Miller T.A. (2004). Osteopontin is a negative regulator of proliferation and differentiation in MC3T3-E1 pre-osteoblastic cells. Bone.

[90] Kohli N., Ho S., Brown S.J., Sawadkar P., Sharma V., Snow M., García-Gareta E. (2018). Bone remodelling in vitro: where are we headed?: -A review on the current understanding of physiological bone remodelling and inflammation and the strategies for testing biomaterials in vitro. Bone.

[91] Li Q., Gao Z., Chen Y., Guan M.-X. (2017). The role of mitochondria in osteogenic, adipogenic and chondrogenic differentiation of mesenchymal stem cells. Protein Cell.

[92] Steinbusch M.M., Cremers A., van Rhijn L.W., Welting T.J. (2016). Unraveling the role of snoRNAs in chondrogenic differentiation. Osteoarthritis Cartilage.

[93] Cabral W.A., Chang W., Barnes A.M., Weis M., Scott M.A., Leikin S., Makareeva E., Kuznetsova N.V., Rosenbaum K.N., Tifft C.J., Bulas D.I., Kozma C., Smith P.A., Eyre D.R., Marini J.C. (2007). Prolyl 3-hydroxylase 1 deficiency causes a recessive metabolic bone disorder resembling lethal/severe osteogenesis imperfecta. Nat. Genet..

[94] Marini J.C., Cabral W.A., Barnes A.M., Chang W. (2007). Components of the collagen prolyl 3-hydroxylation complex are crucial for normal bone development. Cell Cycle.

[95] Nakamura Y.S., Hakeda Y., Takakura N., Kameda T., Hamaguchi I., Miyamoto T., Kakudo S., Nakano T., Kumegawa M., Suda T. (1998). Tyro 3 receptor tyrosine kinase and its ligand, Gas6, stimulate the function of osteoclasts. Stem Cell..

[96] Su J.L., Chiou J., Tang C.H., Zhao M., Tsai C.H., Chen P.S., Chang Y.W., Chien M.H., Peng C.Y., Hsiao M., Kuo M.L., Yenk M.L. (2010). CYR61 regulates BMP-2-dependent osteoblast differentiation through the αvβ3 integrin/integrin-linked kinase/ERK pathway. J. Biol. Chem..

[97] Sen B., Xie Z., Uzer G., Thompson W.R., Styner M., Wu X., Rubin J. (2015). Intranuclear actin regulates osteogenesis. Stem Cell..

[98] Loskutoff D.J. (1991). Regulation of PAI-1 gene expression. Fibrinolysis Proteolysis.

[99] Ermakov S., Toliat M.R., Cohen Z., Malkin I., Altmüller J., Livshits G., Nürnberg P. (2010). Association of ALPL and ENPP1 gene polymorphisms with bone strength related skeletal traits in a Chuvashian population. Bone.

[100] Kim Y.-H., Cho K.-A., Lee H.-J., Park M., Kim H.S., Park J.-W., Woo S.-Y., Ryu K.-H. (2019). Identification of WNT16 as a predictable biomarker for accelerated osteogenic differentiation of tonsil-derived mesenchymal stem cells in vitro. Stem Cell. Int..

