# Supplementary material for: Comparative proteomic analysis of human mesenchymal stromal cell behavior on calcium phosphate ceramics with different osteoinductive potential
Source: Mater Today Bio. 2020 Jun 24;7:100066. doi: 10.1016/j.mtbio.2020.100066 (PMC7334494; doi:10.1016/j.mtbio.2020.100066)
Supplement: Supplementary file 1 — Multimedia component 1 [file mmc1.docx]

**Supplementary Information**

**Validation using Immunohistochemistry**

**Methods**

Cells cultured on TCP and HA particles for 168h in osteogenic medium were fixed using 4% (w/v) paraformaldehyde. After washing the constructs with cold PBS, the ceramics were decalcified with 4% formic acid for 2-3 days at 4 °C. The decalcified samples were dehydrated using an ethanol series of 70, 80, 90, 96 and 100% before being embedded in paraffin wax and cut into 5 μm thick sections. Subsequently, the sections were deparaffinized using xylene and rehydrated in an ethanol series similar to the one used for dehydration in the reverse order. To detect OPN, rabbit anti-OPN (Santa Cruz, Dallas, TX, USA) diluted at the ratio 1:100 in PBS/1%BSA was prepared and incubated on the decalcified sections at room temperature overnight. Alexa Fluor 488-conjugated donkey anti-rabbit IgG (Fisher Scientific) was used to visualize OPN. To visualize nuclei, the cells were stained with DAPI (Sigma) for 10 min at room temperature. Control staining without primary antibody was performed to rule out nonspecific binding of the secondary antibody.

**Results**

The LC-MS/MS data showed an 8-fold higher abundance of OPN on TCP compared to HA after 168 hours (Table S12). The fluorescence microscopy images (Figure S1) confirmed that OPN was present on TCP, whereas no OPN-positive staining was observed on HA. Controls without primary antibody (not shown) showed no signs of nonspecific secondary antibody binding. Note that on TCP, OPN-positive staining was only observed in direct contact with the ceramic surface and in the area between the cells and the material, plausibly due to its high binding affinity for CaP. Such a localization was also observed in an earlier study in which CaP containing materials with adipose-derived mesenchymal stem cells were implanted subcutaneously in nude mice [1]*.* This result substantiates the importance of the interaction between cells and the material surface in the process of osteogenic differentiation.


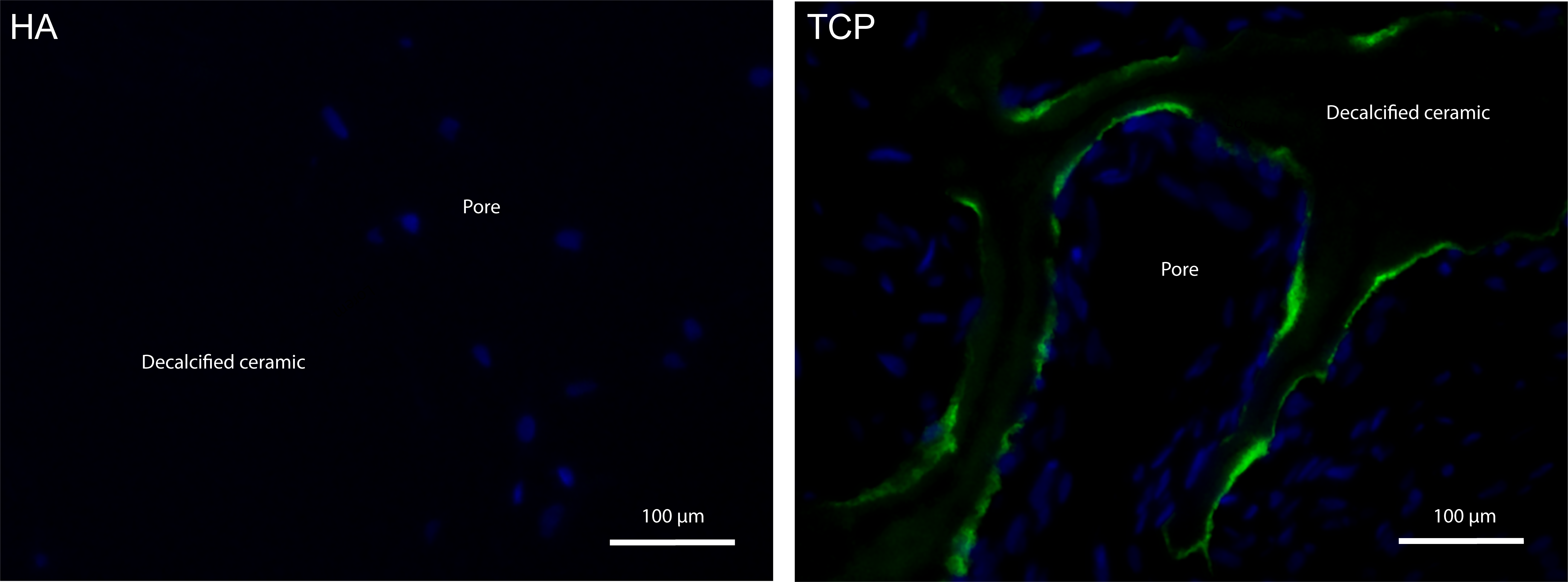


***Figure S1****: Immunofluorescence images showing OPN (Alexa Fluor 488, green) and nuclei (DAPI, blue) in cells cultured for 168 hours on HA (left) and TCP (right) in osteogenic medium. OPN was detected on the surface of TCP. In contrast, no OPN-positive staining was detected on the HA surface.* *Scale bar = 100 µm.*

**References**

[1] H.E. Weiss-Bilka, M.J. Meagher, J.A. Gargac, G.L. Niebur, R.K. Roeder, D.R. Wagner, Mineral deposition and vascular invasion of hydroxyapatite reinforced collagen scaffolds seeded with human adipose-derived stem cells, Biomater. Res. 23 (2019) 15. doi:10.1186/s40824-019-0167-9.

List of Supplementary Tables:

***Table S1:*** *Proteins exclusively adsorbed on TCP after 8h. Relative abundances in arbitrary units.*

***Table S2:*** *Proteins exclusively adsorbed on TCP after 48h. Relative abundances in arbitrary units.*

***Table S3:*** *Proteins exclusively adsorbed on TCP after 168h. Relative abundances in arbitrary units.*

***Table S4:*** *Proteins adsorbed on both HA and TCP after 8h. Relative abundances in arbitrary units.*

***Table S5:*** *Proteins adsorbed on both HA and TCP after 48h. Relative abundances in arbitrary units.*

***Table S6:*** *Proteins adsorbed on both HA and TCP after 168h. Relative abundances in arbitrary units.*

***Table S7:*** *Proteins identified as significantly more abundant after 8 hours in hMSCs cultured on HA compared to hMSCs cultured on TCP. Proteins were selected with a ratio of at least Log2 > 0.38 (at least 1.3-fold change) and maximum adjusted p-value of 0.05.*

***Table S8:*** *Proteins identified as significantly more abundant after 48 hours in hMSCs cultured on HA compared to hMSCs cultured on TCP. Proteins were selected with a ratio of at least Log2 > 0.38 (at least 1.3-fold change) and maximum adjusted p-value of 0.05.*

***Table S9:*** *Proteins identified as significantly more abundant after 168 hours in hMSCs cultured on HA compared to hMSCs cultured on TCP. Proteins were selected with a ratio of at least Log2 > 0.38 (at least 1.3-fold change) and maximum adjusted p-value of 0.05.*

***Table S10:*** *Proteins identified as significantly more abundant after 8 hours in hMSCs cultured on TCP compared to hMSCs cultured on HA. Proteins were selected with a ratio of at least Log2 > 0.38 (at least 1.3-fold change) and maximum adjusted p-value of 0.05.*

***Table S11:*** *Proteins identified as significantly more abundant after 48 hours in hMSCs cultured on TCP compared to hMSCs cultured on HA. Proteins were selected with a ratio of at least Log2 > 0.38 (at least 1.3-fold change) and maximum adjusted p-value of 0.05.*

***Table S12:*** *Proteins identified as significantly more abundant after 168 hours in hMSCs cultured on TCP compared to hMSCs cultured on HA. Proteins were selected with a ratio of at least Log2 > 0.38 (at least 1.3-fold change) and maximum adjusted p-value of 0.05.*

***Table S13:*** *Principal Component Analysis projections for relative abundances of proteins in hMSCs cultured on HA and TCP after 8h, 48h and 168h.*

***Table S14:*** *Top 10 GO Terms with respect to biological process, cellular component, and molecular function, and top 10 Reactome pathways, among proteins upregulated on TCP versus HA after 8h culture.*

***Table S15:*** *Top 10 GO Terms with respect to biological process, cellular component, and molecular function, and top 10 Reactome pathways, among proteins upregulated on HA versus TCP after 8h culture.*

***Table S16:*** *Top 10 GO Terms with respect to biological process, cellular component, and molecular function, and top 10 Reactome pathways, among proteins upregulated on TCP versus HA after 48h culture.*

***Table S17:*** *Top 10 GO Terms with respect to biological process, cellular component, and molecular function, and top 10 Reactome pathways, among proteins upregulated on HA versus TCP after 48h culture.*

***Table S18:*** *Top 10 GO Terms with respect to biological process, cellular component, and molecular function, and top 10 Reactome pathways, among proteins upregulated on TCP versus HA after 168h culture.*

***Table S19:*** *Top 10 GO Terms with respect to biological process, cellular component, and molecular function, and top 10 Reactome pathways, among proteins upregulated on HA versus TCP after 168h culture.*

***Table S20:*** *Top 50 proteins most significantly enriched on osteoinductive ceramics (Dataset 1).*

***Table S21:*** *Proteins linked to BMP-2 and Wnt signalling pathways (from literature).*
